# Supplementary figures and images for: Corrective feedback guides human perceptual decision-making by informing about the world state rather than rewarding its choice
Source: PLoS Biol. 2023 Nov 8;21(11):e3002373. doi: 10.1371/journal.pbio.3002373 (PMC10659185; doi:10.1371/journal.pbio.3002373)

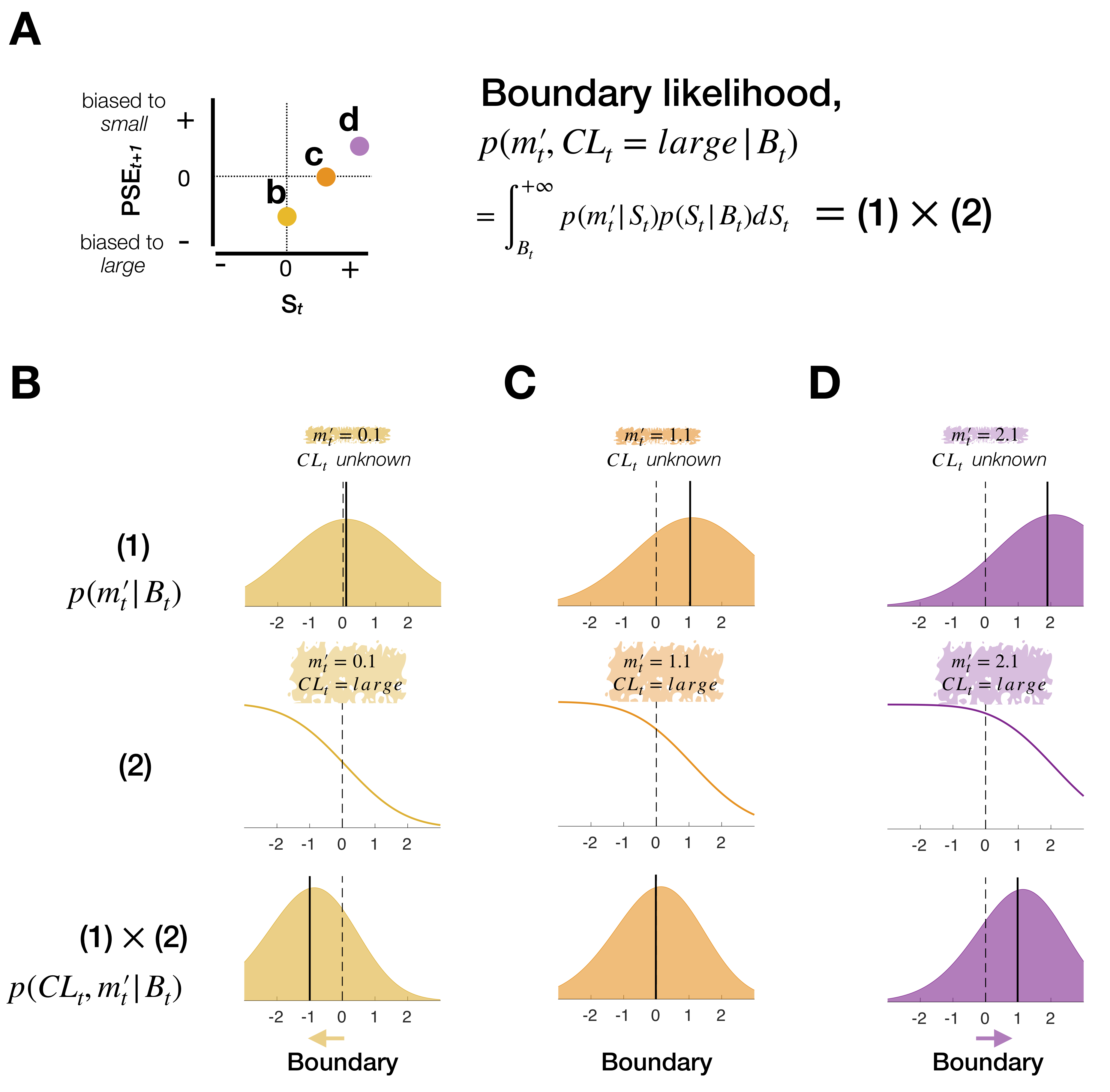

Supplement: S1 Fig — (A) Reversal of subsequent choice bias—expressed in PSE—as a function of sensory evidence strength and boundary inference—expressed in likelihood computation—based on a PDM episode. Left panel: The circles with different colors (indicated by (b-d), which points to the corresponding panels below (B-D)) represent the PSEs associated with the boundary updating for 3 example PDM episodes, where the stimulus (St) varies from 0 to 2 while the choice (Ct) and feedback (Ft) are large and correct, respectively. Right panel: At the core of boundary inference is the computation of the likelihood of the class boundary based on the mnemonic measurement (mt′) and the informed state of the class variable (CLt), where CLt is jointly determined by Ft and Ct (see Materials and methods for the full computation of boundary inference in BMBU). (B-D) The likelihoods of the class boundary given the 3 example PDM episodes defined in (A), where sensory evidence varies from the low (B), to the intermediate (C), and to the high (D) level. To help understand why and how, given the same feedback evidence, the direction of boundary updating reverses as the sensory evidence strengthens, we visualize the boundary likelihoods as a product of 2 functions (Eq 12), indicated by subpanels marked as (1) and (2). Top row: As indicated by (1), we plot each boundary likelihood when only the mnemonic measurement is considered, assuming that no feedback is provided. Note that these likelihood functions are centered around the values of mt′, by attracting the class boundary toward themselves, driving a shift towards the large side (i.e., positive side on the boundary axis). Middle-Bottom rows: When the feedback evidence is given—i.e., when the informed state of CLt is revealed as large—in addition to the mnemonic measurement, an additional piece of information about the class boundary arises. As indicated by (1) × (2), we plot each boundary likelihood (defined in (A)). As indicated by (2), we plot each functi [file pbio.3002373.s001.tif]

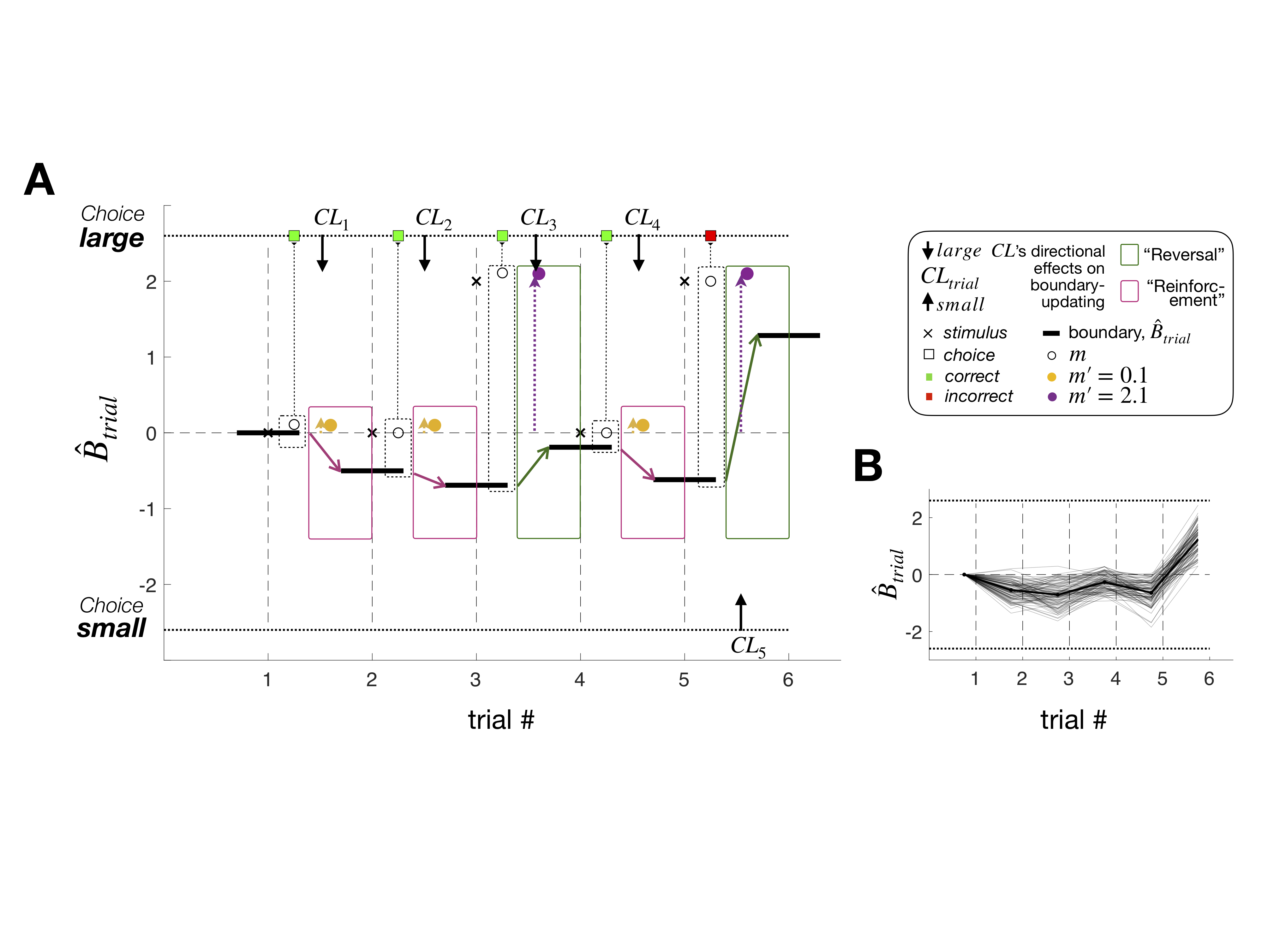

Supplement: S2 Fig — (A) An example trial history to show how a temporal trajectory of the class boundary inferred by BMBU. For example, at trial #1 (x-axis), a physical stimulus (symbol x) was 0, a sensory measurement (symbol o) was a positive value when the boundary belief (solid black bar; y-axis) was centered at 0. BMBU’s choice was large (symbol square on the top of y-axis), and correct feedback (same square filled with green color) was provided, which indicates that the class variable at trial #1 CL1 was large (arrow’s direction indicates the effect of the trial class variable on the subsequent boundary-updating). BMBU updates one’s belief based on evidence from stimulus (colored symbol o) and feedback (CL1), available at the time of boundary-updating. To illustrate cases where the bias reversal we defined in Fig 3D in the main text happen and do not happen, same examples were intentionally used as those we used in S1 Fig where we further detailed on the model’s mechanisms. Depending on colors, sensory evidence is weak (yellow symbol o) or strong (purple symbol o), which leads to whether or not the reversal happens. Trial cases featured in a red box indicates that the “Reinforcement” principle is held (predicting subsequent choices to repeat large choice) while those featured in a green box indicates that the “Reversal” happens (predicting subsequent choices to reverse the previously made large choice). (B) Temporal trajectories of the class boundary when the same 6-trial sequence of physical stimuli in (A) was simulated for 100 times. This means different m and m′ were realized. The data underlying this figure (A, B) can be found in S1 Data. (TIF) [file pbio.3002373.s002.tif]

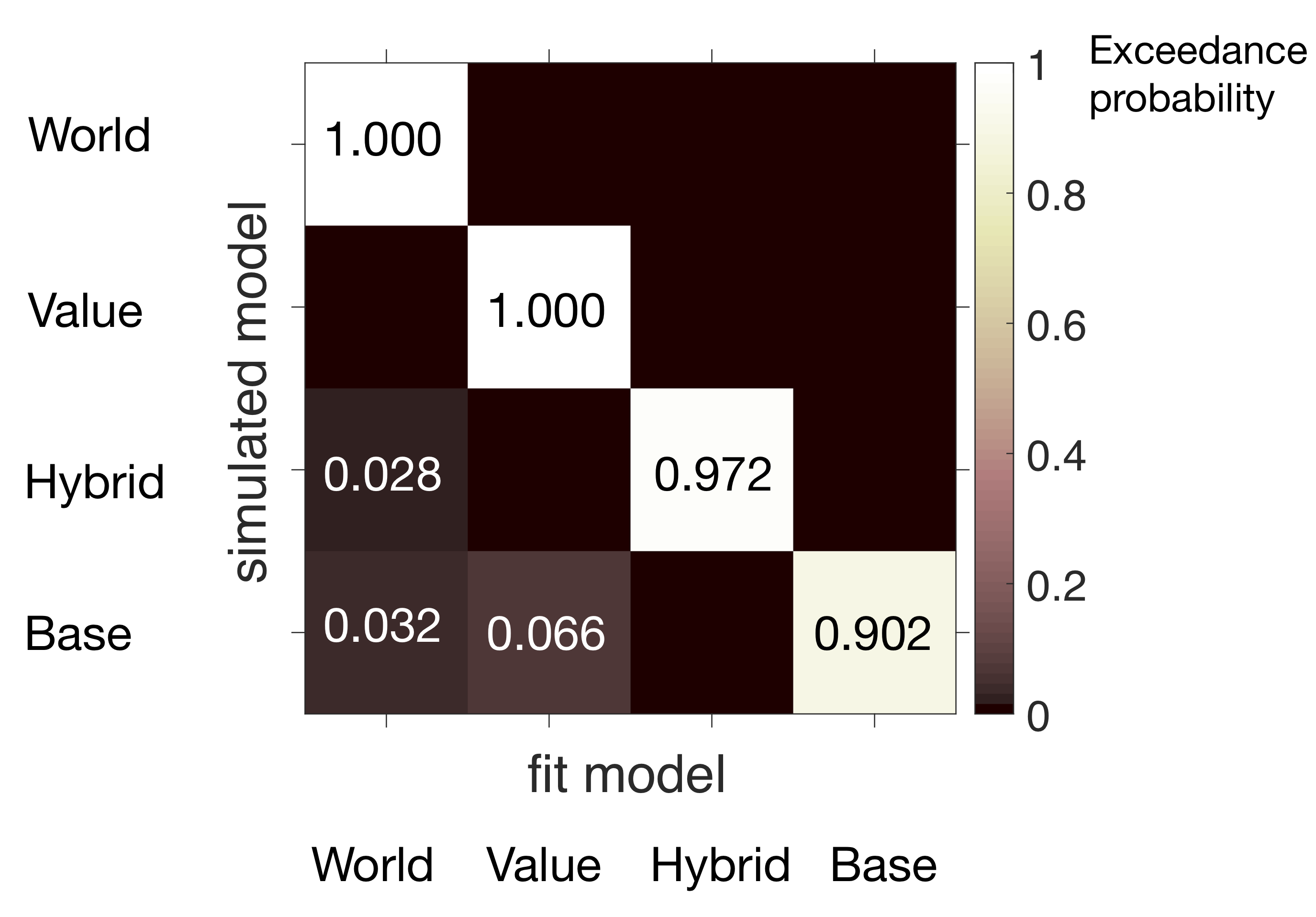

Supplement: S3 Fig — Each square represents exceedance probability pexc from model recovery procedure. The “ground-truth” model to simulate synthetic behavior was correctly recovered with pexc >0.9 for all 4 models considered in the study. The light shade of the diagonal squares indicates that the ground-truth model was the best-fitting model, leading to a successful model recovery. Numerical values can also be found in S1 Data. (TIF) [file pbio.3002373.s003.tif]

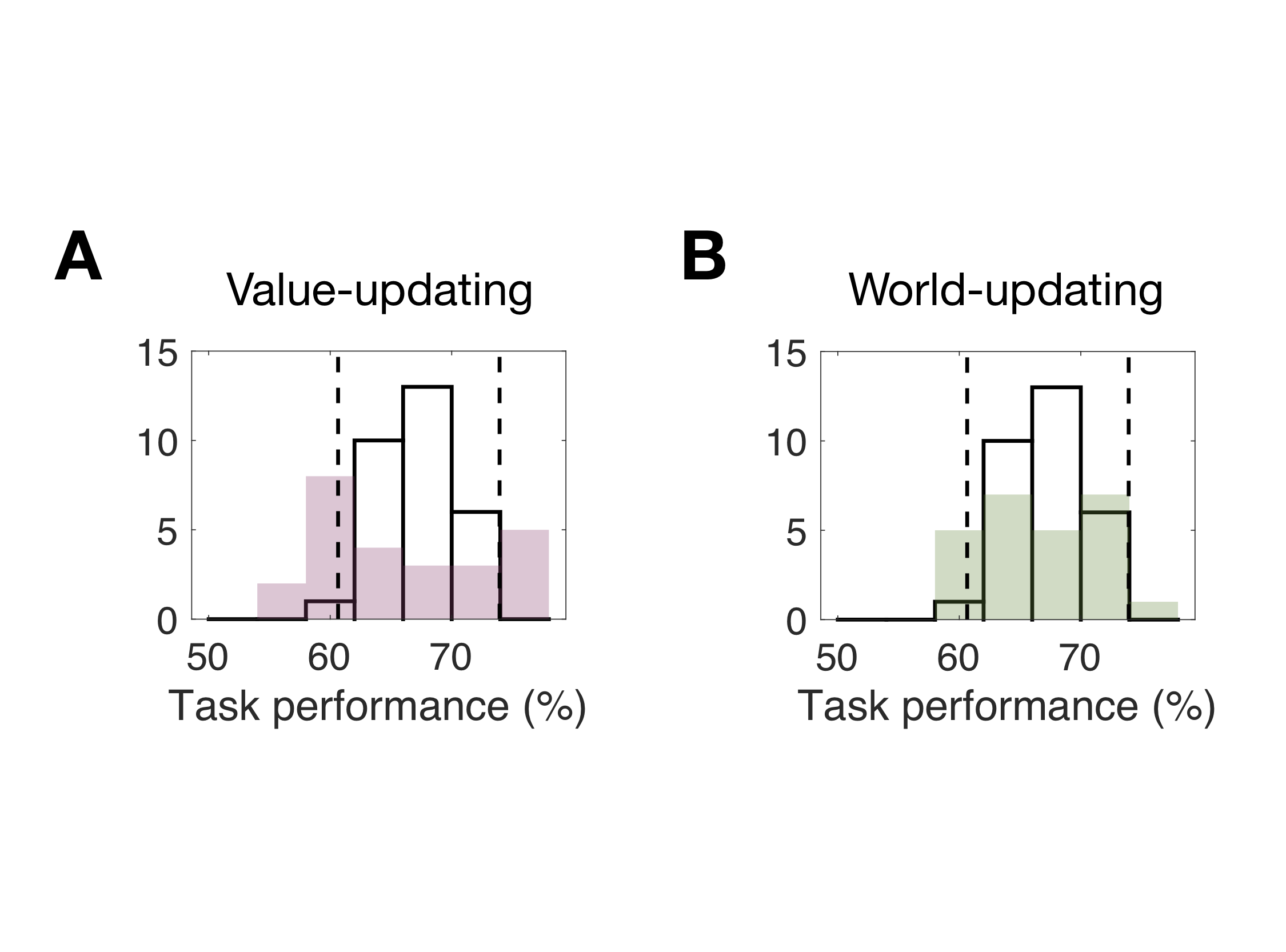

Supplement: S4 Fig — (A, B) Across-individual distributions of the classification accuracy of the belief-based RL model (A) and BMBU (B) overlaid on those of the human participants. The models’ choices were generated via ex ante simulations with a specific set of model parameters (Table A in S1 Appendix), the results of which are depicted in Figs 4 and 5. The classification accuracy is measured by calculating the percentage of the trials in which the choice matched the feedback used in the actual experiment. The empty bars correspond to the histogram of human performances, the range of which is demarcated by the dashed vertical lines ([min, max] = [60.65%, 73.94%]). The average human classification accuracy was 67.85%. (A) Comparison of classification accuracy between the belief-based RL model’s simulation (red color) and the human choices. The model’s ex ante simulation accuracy was not different from the human accuracy (t(53) = 1.4429, P = 0.1549; Null hypothesis: model’s performance vector and humans’ performance vector come from populations with equal means, unpaired two-tailed t test). (B) Comparison of classification accuracy between BMBU’s simulation (green color) and the human choices. The model’s ex ante simulation accuracy was not different from the human accuracy (t(53) = 0.9707, P = 0.3361, unpaired two-tailed t test). There was no significant difference in classification accuracy between the value-updating model and BMBU (t(48) = 0.5733, P = 0.5691, unpaired two-tailed t test). The data underlying this figure (A, B) can be found in S1 Data. (TIF) [file pbio.3002373.s004.tif]

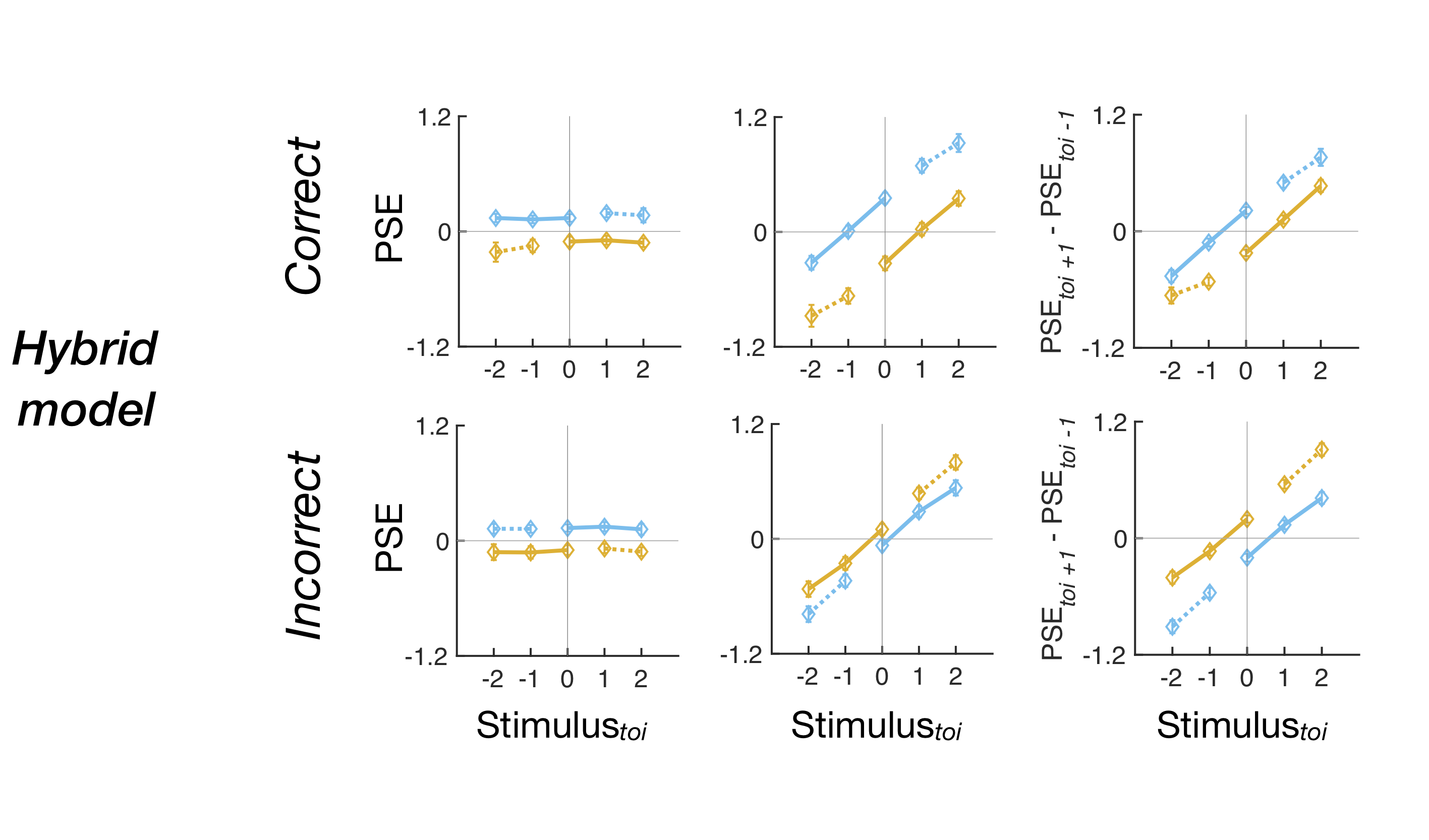

Supplement: S5 Fig — Top and bottom rows in each panel show the PSEs associated with the toi episodes involving correct and incorrect feedback at toi. Symbols with error bars, mean ± SEM across the 30 model agents, which correspond to their 30 human partners. The colors of the symbols and lines label choices (blue: small and yellow: large). The data underlying this figure can be found in S1 Data. (TIF) [file pbio.3002373.s005.tif]

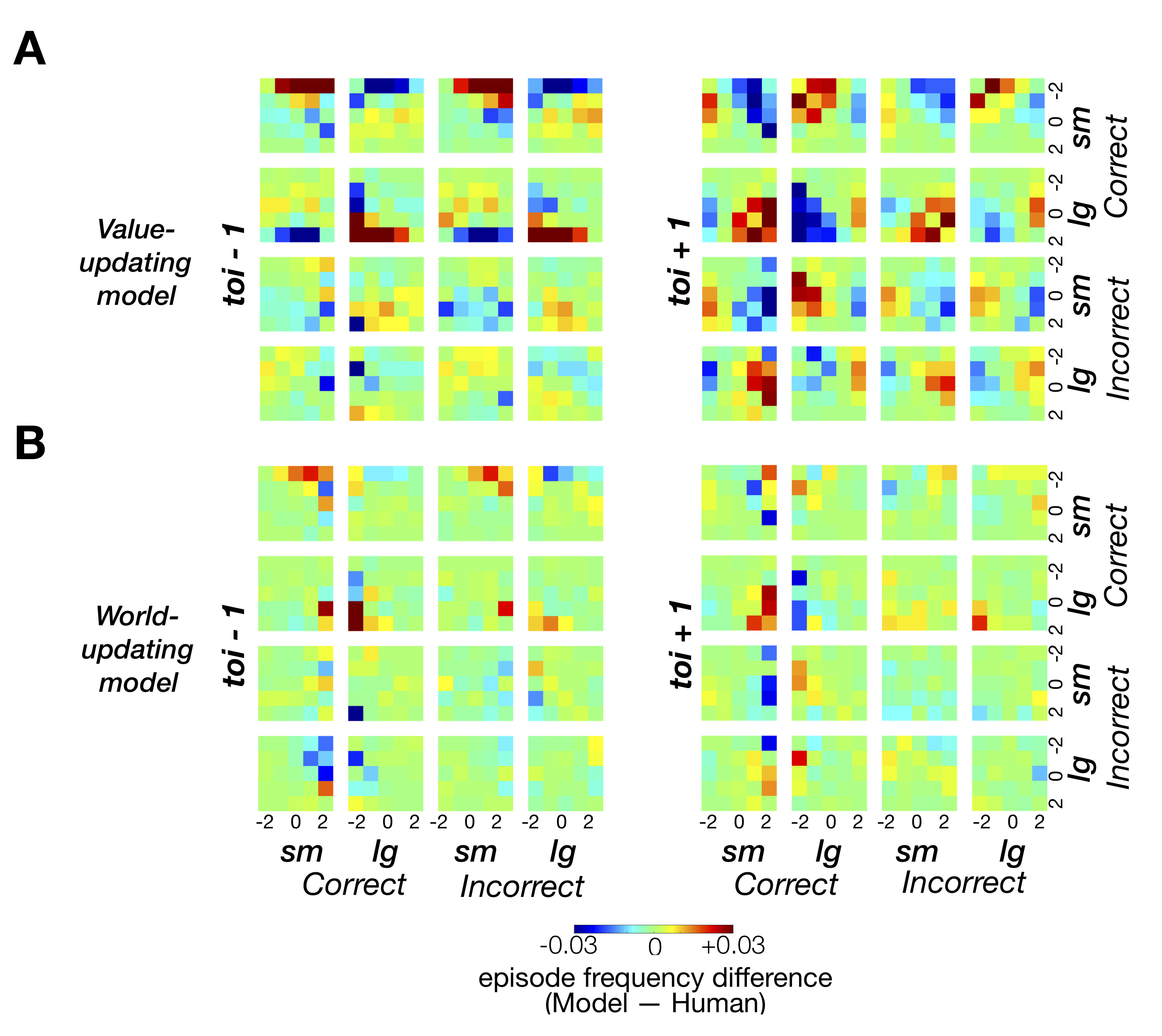

Supplement: S6 Fig — Maps of frequency deviations of the value-updating (A) and world-updating (B) model agents’ classifications in the ex post simulations from the human decision-makers in the retrospective (left) and prospective (right) history effects. Each cell represents a pair of PDM episodes, as specified by the column and row labels. At each cell, the color represents how much the episode frequency observed in the model agents deviates from that observed in the corresponding human decision-makers. The results of statistical tests on these deviations are summarized in Fig 7E and 7F. The data underlying this figure (A, B) can be found in S1 Data. (TIF) [file pbio.3002373.s006.tif]

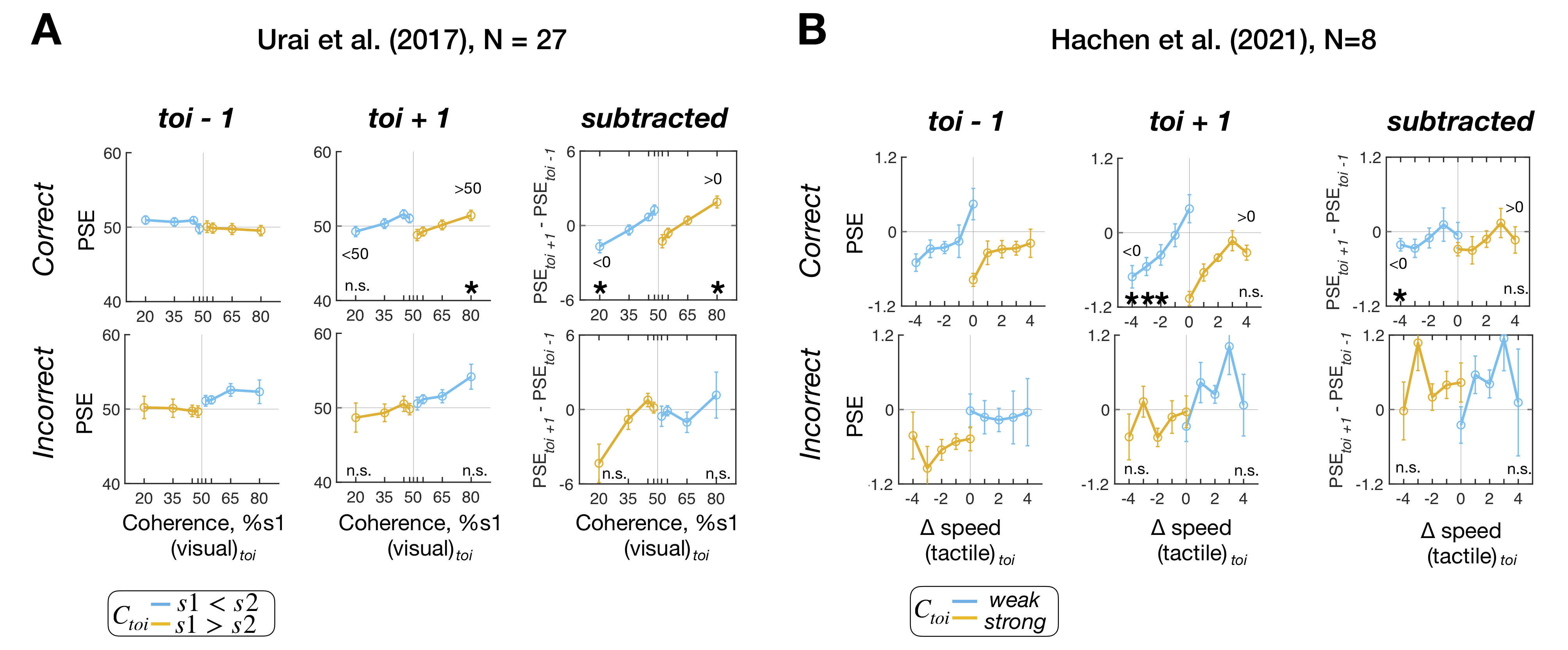

Supplement: S7 Fig — Retrospective (left columns), prospective (middle columns), and subtractive (right columns) history effects in PSE for the human classification performances of Urai and colleagues’ work [37] (A) and Hachen and colleagues’ work [31] (B). (A, B) We downloaded both publicly available datasets, analyzed them in the same way that we analyzed human observers in our work, and plotted the results in the same format used for Fig 7A. Top and bottom rows in each panel show the PSEs associated with the toi episodes involving correct and incorrect feedback. Symbols with error bars, mean ± SEM across human observers. The colors of the symbols and lines label choices (blue: small and yellow: large). The overall patterns of the PSEs plotted here appear similar to those plotted in Fig 7A, displaying the reversals in direction of stimulus-dependent feedback effects. When the same statistical tests used in our work were carried out, some of the data points at the stimuli with strong sensory evidence at toi significantly deviated from zero in the direction opposite to the feedback effect predicted by the value-updating scenario, as indicated by the asterisks. (A) Sequential features of human observers (N = 27) analyzed in our way from human dataset that once had been published [37], which is openly available (http://dx.doi.org/10.6084/m9.figshare.4300043), then analyzed in the previous study [9]. In this study, the participants performed a binary classification task on the difference in motion coherence by sorting the pairs of random-dot-kinematogram stimuli shown in 2 intervals (s1 and s2) into one of the 2 classes (“s1<s2” vs. “s1>s2”) over consecutive trials. The presented stimuli were taken from 3 sets of difficulty levels (the difference between motion coherence of the test and the reference stimulus; easy: [2.5, 5, 10, 20, 30], medium: [1.25, 2.5, 5, 10, 30], hard: [0.625, 1.25, 2.5, 5, 20]). As done in the original study [9], we binned the trials into 8 levels by merging the tri [file pbio.3002373.s007.tif]
