# Supplementary material for: Corrective feedback guides human perceptual decision-making by informing about the world state rather than rewarding its choice
Source: PLoS Biol. 2023 Nov 8;21(11):e3002373. doi: 10.1371/journal.pbio.3002373 (PMC10659185; doi:10.1371/journal.pbio.3002373)
Supplement: S1 Appendix — Supplemental details (Text) on additional model specifications of BMBU are provided. Supplementary tables (A-D Tables) to support the Results section are provided. Table A. Parameters used for ex ante simulations. Table B. Parameters recovered from fitting the main models, world-updating and value-updating models, to human choices (N = 30). Table C. Parameters recovered from fitting the rest of the models to human choices (N = 30). Table D. Statistical results on model behavior versus human behavior in terms of PSE measures. (DOCX) [file pbio.3002373.s008.docx]

Corrective feedback guides human perceptual decision-making

by informing about the world state rather than rewarding its choice

S1 Appendix

Supporting details

Hyang-Jung Lee^1^, Heeseung Lee^1^, Chae Young Lim^2^, Issac Rhim^3^, Sang-Hun Lee^1^

^1^Department of Brain and Cognitive Sciences, Seoul National University, Seoul, South Korea

^2^Department of Statistics, Seoul National University, Seoul, South Korea

^3^Institute of Neuroscience, University of Oregon, Eugene, Oregon, USA

*Correspondence: visionsl@snu.ac.kr

Supplemental details (Text) *on additional model specifications of BMBU*

Here, we will elaborate on "**World-updating model**", a subsection found in the **Materials and methods** of the main text. We will provide more details on the boundary-updating operation of BMBU by explaining how Equation 10 is derived from Equation 9 in the main text.

Equation 8 in the main text defines how the likelihood function of the class boundary $B_{t}$ given two pieces of evidence, the mnemonic measurement $m_{t}^{'}$—i.e., noisy memory recall of the sensory measurement $m_{t}$ in working memory—and the state of the class variable ${CL}_{t}$ informed by feedback. The boundary likelihood, which represents the probabilities of those two observations under any hypothesized state of $B_{t}$, is calculated through integration (marginalization) over all possible states of the unknown variable $S_{t}$, and can be re-written as:

$$\begin{aligned} p\left( m_{t}^{'}, {CL}_{t} | B_{t} \right)=\int p\left( m_{t}^{'} | S_{t} \right)p\left( {CL}_{t} | S_{t}{, B}_{t} \right)p\left( S_{t}{|B}_{t} \right)dS_{t}=\int L_{m_{t}^{'}}\left( S_{t} \right)L_{{CL}_{t}}\left( S_{t}{, B}_{t} \right)p\left( S_{t}{|B}_{t} \right)dS_{t}, \#\left( S1 \right) \end{aligned}$$

where each integrand is elaborated in the following.

The factor $p\left( m_{t}^{'} | S_{t} \right)$ in Equation S1 corresponds to the information about the stimulus offered by the mnemonic measurement $m_{t}^{'}$. The likelihood of any hypothetical state of $S_{t}$ given $m_{t}^{'}$ refers to the probability of $m_{t}^{'}$ if that hypothetical state is true. Here, we denote $p\left( m_{t}^{'} | S_{t} \right)$ by a likelihood function, $L_{m_{t}^{'}}\left( S_{t} \right).$

Through marginalization over $m_{t}$ from the learned generative model, which is described by Equations 3-6 in the main text, $L_{m_{t}^{'}}\left( S_{t} \right)\equiv p\left( m_{t}^{'} | S_{t} \right)$ can be expressed in a form of the Gaussian function, as follows:

$$\begin{aligned} p\left( m_{t}^{'} | S_{t} \right)=\int p\left( m_{t}^{'}|m_{t} \right)p\left( m_{t}|S_{t} \right)dm_{t}=\int N\left( m_{t}^{'};m_{t}, \sigma_{m^{'}}^{2} \right)N\left( m_{t};S_{t}, \sigma_{m}^{2} \right){dm}_{t} \\ =\int\frac{1}{\sqrt{2\pi\sigma_{m^{'}}^{2}}}e^{-\frac{\left( m_{t}^{'}-m_{t} \right)^{2}}{2\sigma_{m^{'}}^{2}}}\frac{1}{\sqrt{2\pi\sigma_{m}^{2}}}e^{-\frac{\left( m_{t}-S_{t} \right)^{2}}{2\sigma_{m}^{2}}}{dm}_{t}=N\left( m_{t}^{'};S_{t}, \sigma_{m^{'}}^{2}+\sigma_{m}^{2} \right), \#\left( S2 \right) \end{aligned}$$

where the expressions $p\left( m_{t}^{'}|m_{t} \right)p\left( m_{t}|S_{t} \right)$ are substituted by the two Gaussian noise distributions as defined in the learned generative model (Equation 5-6 in the main text) and integrated over the possible states of the sensory measurement variable $m_{t}$, which is now unknown—inaccessible any longer. Thus, we find that $L_{m_{t}^{'}}\left( S_{t} \right)$, the likelihood of $S_{t}$, follows $N\left( S_{t};m_{t}^{'}, \sigma_{m^{'}}^{2}+\sigma_{m}^{2} \right)$.

Next, the factor $p\left( {CL}_{t} | S_{t}{, B}_{t} \right)$ in Equation S1 corresponds to the information about the stimulus and the class boundary offered by the state of the class variable ${CL}_{t}$ informed by feedback, either $small$ or $large$. The likelihood of any hypothetical joint states of $S_{t}$ and$B_{t}$ given ${CL}_{t}$ refers to the probability of ${CL}_{t}$ if that hypothetical state is true. Here, we denote $p\left( {CL}_{t} | S_{t}{, B}_{t} \right)$ by a likelihood function, $L_{{CL}_{t}}\left( S_{t}{, B}_{t} \right).$ $L_{{CL}_{t}}\left( S_{t}{, B}_{t} \right)$ is determined depending on the inequality between $S_{t}$ and$B_{t}$: in the case of ${CL}_{t}=small$,

$$\begin{aligned} L_{{CL}_{t}=small}\left( S_{t}{, B}_{t} \right)=p\left( {CL}_{t}=small | S_{t}{, B}_{t} \right)=\left\{ \begin{aligned} 1, &S_{t}<B_{t} \\ 0, &S_{t}>B_{t}\#\#\# \\ 0.5, &S_{t}=B_{t} \#\#\#\#\#\#\# \end{aligned} \right.\#\left( S3 \right) \end{aligned}$$

; in the case of ${CL}_{t}=large$,

$$\begin{aligned} L_{{CL}_{t}=large}\left( S_{t}{, B}_{t} \right)=p\left( {CL}_{t}=large | S_{t}{, B}_{t} \right)=\left\{ \begin{aligned} 0, &S_{t}<B_{t} \\ 1, &S_{t}>B_{t} \\ 0.5, &S_{t}=B_{t} \end{aligned} \right. .\#\left( S4 \right) \end{aligned}$$

Now, let us get back to Equation S1 and denote the boundary likelihood function in the case of ${CL}_{t}=small$ (abbreviated as $s$) by $L_{m_{t}^{'},{CL}_{t}=s}\left( B_{t} \right)$, which can further be decomposed into two integrals with finite limits (as similarly done for Equation 9 in the main text), as follows:

$$L_{m_{t}^{'},{CL}_{t}=s}\left( B_{t} \right)\equiv p\left( m_{t}^{'}, {CL}_{t}=s | B_{t} \right)$$

$=\int_{S_{t}=-\infty}^{S_{t}=B_{t}} p\left( m_{t}^{'} | S_{t} \right)p\left( {CL}_{t}=s | S_{t}{, B}_{t} \right)p\left( S_{t}{|B}_{t} \right)dS_{t}+\int_{{S_{t}=B}_{t}}^{S_{t}=+\infty} p\left( m_{t}^{'} | S_{t} \right)p\left( {CL}_{t}=s | S_{t}{, B}_{t} \right)p\left( S_{t}{|B}_{t} \right)dS_{t},$

which can be rewritten as follows:

$$=\lim_{r\to\infty, b\to B_{t}^{-}} \int_{-r}^{b} p\left( m_{t}^{'} | S_{t} \right)p\left( {CL}_{t}=s | S_{t},B_{t} \right)p\left( S_{t}|B_{t} \right)dS_{t}+\lim_{r\to\infty, b\to B_{t}^{+}} \int_{b}^{r} p\left( m_{t}^{'} | S_{t} \right)p\left( {CL}_{t}=s | S_{t},B_{t} \right)p\left( S_{t}|B_{t} \right)dS_{t}.$$

$\begin{aligned} \#\left( S5 \right) \end{aligned}$

Since the last term on the right-hand side of Equation S5 becomes zero by Equation S3 (for any value ranges of $S_{t}$ larger than $B_{t}$, $p\left( {CL}_{t}=s | S_{t},B_{t} \right)=0$), the boundary likelihood function in the case of ${CL}_{t}=small$ is reduced as follows:

$$\begin{aligned} L_{m_{t}^{'},{CL}_{t}=s}\left( B_{t} \right)\equiv p\left( m_{t}^{'}, {CL}_{t}=s | B_{t} \right)=\int_{-\infty}^{B_{t}} p\left( m_{t}^{'} | S_{t} \right)p\left( S_{t}{|B}_{t} \right)dS_{t}.\#\left( S6 \right) \end{aligned}$$

From Equation 3 in the main text and Equation S2, we know the distributions $p\left( S_{t}{|B}_{t} \right)$ and$p\left( m_{t}^{'} | S_{t} \right)$, respectively. Substituting the expression for these distributions gives:

$$L_{m_{t}^{'},{CL}_{t}=s}\left( B_{t} \right)$$

$=\int_{-\infty}^{B_{t}} \frac{1}{\sqrt{2\pi\left( \sigma_{m^{'}}^{2}+\sigma_{m}^{2} \right)}}e^{-\frac{\left( m_{t}^{'}-S_{t} \right)^{2}}{2\left( \sigma_{m^{'}}^{2}+\sigma_{m}^{2} \right)}}\frac{1}{\sqrt{2\pi\sigma_{S}^{2}}}e^{-\frac{\left( S_{t}-B_{t} \right)^{2}}{2\sigma_{S}^{2}}}dS_{t}=\frac{1}{\sqrt{2\pi\left( \frac{\sigma_{M}^{2}\sigma_{S}^{2}}{\left( \sigma_{M}^{2}+\sigma_{S}^{2} \right)} \right)}}\int_{-\infty}^{B_{t}} e^{-\frac{\left( S_{t}-\frac{B_{t}\sigma_{M}^{2}+m_{t}^{'}\sigma_{S}^{2}}{\sigma_{M}^{2}+\sigma_{S}^{2}} \right)^{2}}{2\frac{\sigma_{M}^{2}\sigma_{S}^{2}}{\left( \sigma_{M}^{2}+\sigma_{S}^{2} \right)}}}dS_{t}\times\frac{1}{\sqrt{2\pi\left( \sigma_{M}^{2}+\sigma_{S}^{2} \right)}}e^{-\frac{\left( B_{t}-m_{t}^{'} \right)^{2}}{2\left( \sigma_{M}^{2}+\sigma_{S}^{2} \right)}}$,

$\begin{aligned} \#\left( S7 \right) \end{aligned}$

where $\sigma_{M}^{2}=\sigma_{m^{'}}^{2}+ \sigma_{m}^{2}$. Equation S7 is equivalent to Equation 11 in the main text.

To aid in intuitive comprehension of $L_{m_{t}^{'},{CL}_{t}=s}\left( B_{t} \right)$, we can express it as the product of two terms from Equation S7 by rewriting it as follows:

$$\begin{aligned} L_{m_{t}^{'},{CL}_{t}=s}\left( B_{t} \right)=F_{X}\left( B_{t} \right)\mathcal{L}_{m_{t}^{'}}\left( B_{t} \right),\#\left( S8 \right) \end{aligned}$$

where $F_{X}$ denotes the first term on the right-hand side of Equation S7 (to be detailed in Equation S10), and $\mathcal{L}_{m_{t}^{'}}$ denotes the second term on the right-hand side of Equation S7 (to be detailed in Equation S9), which equals to the likelihood function for $B_{t}$ given $m_{t}^{'}$ defined under the assumption that no ${CL}_{t}$ variable exists in the generative process. To make this point explicit, we used a different likelihood notation $\mathcal{L}$ from the notation $L$ used throughout the paper.

Note that, according to the learned generative model in our study, the decision-maker acquires the knowledge about $B_{t}$ by evaluating how probable the two observations, $m_{t}^{'}$ (sensory evidence in memory; simply as sensory evidence, hereinafter) and ${CL}_{t}$ (feedback evidence), are for each possible value of $B_{t}$. Contrastingly, $\mathcal{L}_{m_{t}^{'}}\left( B_{t} \right)$ in Equation S8 can be considered as the information about $B_{t}$ solely based on the sensory evidence by computing $p\left( m_{t}^{'} | B_{t} \right)$. Thus, $\mathcal{L}_{m_{t}^{'}}\left( B_{t} \right)$ represents the “sensory influence” that drives the boundary update, by pushing the joint boundary likelihood $L_{m_{t}^{'},{CL}_{t}=s}\left( B_{t} \right)$towards $m_{t}^{'}$ on the $B_{t}$axis (see the first row in S1B-D Fig), as follows:

$$\begin{aligned} \mathcal{L}_{m_{t}^{'}}\left( B_{t} \right)=\frac{1}{\sqrt{2\pi\left( \sigma_{M}^{2}+\sigma_{S}^{2} \right)}}e^{-\frac{\left( B_{t}-m_{t}^{'} \right)^{2}}{2\left( \sigma_{M}^{2}+\sigma_{S}^{2} \right)}}=N\left( B_{t};m_{t}^{'}, \sigma_{M}^{2}+\sigma_{S}^{2} \right).\#\left( S9 \right) \end{aligned}$$

On the other hand, the term $F_{X}\left( B_{t} \right)$ in Equation S8 can be considered as the information about $B_{t}$ jointly based on the feedback and sensory evidence, while excluding the aforementioned “sensory influence”, i.e., divided by $\mathcal{L}_{m_{t}^{'}}\left( B_{t} \right)$. Let $X$ be a random variable with cumulative distribution function (CDF) $F_{X}$ by

$$\begin{aligned} F_{X}\left( B_{t} \right)=\int_{-\infty}^{B_{t}} f_{X}\left( S_{t} \right)dS_{t}, \#\left( S10 \right) \end{aligned}$$

where the probability density function of $X$, denoted by $f_{X}\left( x \right)=N\left( x;\frac{B_{t}\sigma_{M}^{2}+m_{t}^{'}\sigma_{S}^{2}}{\sigma_{M}^{2}+\sigma_{S}^{2}}, \frac{\sigma_{M}^{2}\sigma_{S}^{2}}{\left( \sigma_{M}^{2}+\sigma_{S}^{2} \right)} \right)$. Since this term is derived specifically for ${CL}_{t}=small$, we can interpret this term as the “feedback influence” that drives the boundary update with a CDF multiplied, by pushing the boundary likelihood $L_{m_{t}^{'},{CL}_{t}=s}\left( B_{t} \right)$ in a more positive direction on the $B_{t}$axis. Owing to this contribution by the feedback evidence, the boundary likelihood $L_{m_{t}^{'},{CL}_{t}=s}\left( B_{t}=b \right)$ for a given value $b$ of $B_{t}$ would support that $b>m_{t}^{'}$ is more plausible than $b<m_{t}^{'}$ for the current state of the class boundary that generated $m_{t}^{'}$ and ${CL}_{t}=small$. This aligns with the intuition from the temperature example described in the main text (Fig 1C).

Similarly, from Equations S5-S7, we derive the boundary likelihood for the case ${CL}_{t}=large$:

$$\begin{aligned} L_{m_{t}^{'},{CL}_{t}=l}\left( B_{t} \right)=\int_{B_{t}}^{\infty} p\left( m_{t}^{'} | S_{t} \right)p\left( S_{t}{|B}_{t} \right)dS_{t}={(1-F}_{X}\left( B_{t} \right))\mathcal{L}_{m_{t}^{'}}\left( B_{t} \right), \#\left( S11 \right) \end{aligned}$$

Contrary to the ${CL}_{t}=small$ case, the boundary likelihood $L_{m_{t}^{'},{CL}_{t}=l}\left( B_{t}=b \right)$ would support that $b<m_{t}^{'}$ is more plausible than $b>m_{t}^{'}$ for $B_{t}$ that generated $m_{t}^{'}$ and ${CL}_{t}=large$, since the multiplication is performed, instead with a complementary CDF (see the second row in S1B-D Fig).

Supplementary tables (A-D Tables) *to support the Results section*

**Table A. Parameters used for *ex ante* simulations.**

| World |  | Value |  |
| --- | --- | --- | --- |
| $\sigma_{m}$ | 25 levels  $[0.15, 3.27]$ | $\sigma_{m}$ | 25 levels  $[0.15, 3.27]$ |
| $\mu_{0}$ | $0$ | $\mu_{0}$ | $0$ |
| $\sigma_{0}$ | $5$ | $\alpha$ | $0.35$ |
| $\sigma_{s}$ | $1.5811$ | $\beta$ | $5$ |
| $\sigma_{m^{'}}$ | $2.5$ | $V_{init}$ | $0.5$ |
| $\sigma_{diffusion}$ | $0.8$ |  |  |

**Table B. Parameters recovered from fitting the main models, world-updating and value-updating models, to human choices (**N=30**).**

| World | Mean  (standard deviation) | Value | Mean  (standard deviation) |
| --- | --- | --- | --- |
| $\sigma_{m}$ | $0.86$  $(0.46)$ | $\sigma_{m}$ | $1.20$  $(0.30)$ |
| $\mu_{0}$ | $0.04$  $(0.62)$ | $\mu_{0}$ | $0.02$  $(0.43)$ |
| $\sigma_{0}$ | $4.27$  $(1.22)$ | $\alpha$ | $0.17$  $(0.20)$ |
| $\sigma_{s}$ | $0.84$  $(0.92)$ | $\beta$ | $7.43$  $(7.01)$ |
| $\sigma_{m^{'}}$ | $3.70$  $(1.00)$ | $V_{init}$ | $0.92$  $(0.16)$ |
| $\sigma_{diffusion}$ | $3.20$  $(1.57)$ |  |  |

**Table C. Parameters recovered from fitting the rest of the models to human choices (**N=30**).**

| Hybrid | Mean  (standard deviation) | Fixed | Mean  (standard deviation) | Base | Mean  (standard deviation) |
| --- | --- | --- | --- | --- | --- |
| $\sigma_{m}$ | $0.68$  $(0.33)$ | $\sigma_{m}$ | $1.54$  $(0.39)$ | $\sigma_{m}$ | $1.90$  $(0.50)$ |
| $\mu_{0}$ | $0.01$  $(0.61)$ | $\mu_{0}$ | $0.02$  $(0.31)$ |  |  |
| $\sigma_{0}$ | $3.55$  $(1.28)$ |  |  |  |  |
| $\sigma_{s}$ | $0.86$  $(1.03)$ |  |  |  |  |
| $\sigma_{m^{'}}$ | $3.84$  $(1.22)$ |  |  |  |  |
| $\sigma_{diffusion}$ | $3.77$  $(1.87)$ |  |  |  |  |
| $\alpha$ | $0.15$  $(0.11)$ |  |  |  |  |
| $\beta$ | $6.96$  $(2.83)$ |  |  |  |  |
| $V_{init}$ | $0.882$  $(0.15)$ |  |  |  |  |

**Table D. Statistical results on model behavior versus human behavior in terms of PSE measures.**

| Model vs.  Data | *toi-1*  (20 conditions) | | | | | *toi+1*  (20 conditions) | | | | (*toi+1) - (toi-1)*  (20 conditions) | | | | Total  (60 conditions) | |
| --- | --- | --- | --- | --- | --- | --- | --- | --- | --- | --- | --- | --- | --- | --- | --- |
| Statistical difference | n.s. | | | significant | | n.s. | | significant | | n.s. | | significant | | n.s.  # conditions | |
| Test type | *t* | | *w* | *t* | *w* | *t* | *w* | *t* | *w* | *t* | *w* | *t* | *w* | *t* | *w* |
| Value | (13) | (13) | | *(1)  **(2)  ***(4) | *(1)  **(5)  ***(1) | (10) | (9) | *(1)  **(1)  ***(8) | *(3)  **(3)  ***(5) | (8) | (9) | *(1)  **(1)  ***(10) | **(6)  ***(5) | (31) | (31) |
| World | (20) | (20) | | . | . | (20) | (20) | . | . | (20) | (20) | . | . | (60) | (60) |
| Hybrid | (20) | (20) | | . | . | (17) | (19) | *(3) | *(1) | (19) | (20) | *(1) | . | (56) | (59) |

Test type, *t:*  Paired t-test

Test type, *w*: Wilcoxon signed-rank test

*: *P*<0.00083, Bonferroni-corrected threshold

**: *P*<0.000167,

***: *P*<0.0000167
