# Supplementary material for: Corrective feedback guides human perceptual decision-making by informing about the world state rather than rewarding its choice
Source: PLoS Biol. 2023 Nov 8;21(11):e3002373. doi: 10.1371/journal.pbio.3002373 (PMC10659185; doi:10.1371/journal.pbio.3002373)
Supplement: Attachment — Submitted filename: RespToReviewers_Reply_Lee_et_al.docx [file pbio.3002373.s011.docx]

[Responses to Reviews]

We thank the reviewers for acknowledging the contributions of our study and providing constructive feedback on the previous version of our manuscript. We have thoroughly studied their comments, addressed each point, and made necessary revisions to the manuscript, as summarized below.

To aid in clarity and referencing, we will refer to the major comments of the reviewers as C#-n and their minor comments as M#-n, where # and n represent the reviewer’s identity and the order, respectively. Whenever a comment needed to be addressed separately, we marked it with a letter (e.g., C1-1 was divided into two separate parts, C1-1a and C1-1b).

=====================================================================

**Comments from Reviewer #1:**

“The authors investigate the origins of correcting feedback in perceptual decision making by using computational modelling and human experiments. When comparing the predictions of a value-based model vs a world's inference model, they find that the second type explains the data much better. Most importantly, the two models different by the type of qualitative predictions that they make: the world's inference model is the only one that can explain a bias reversal that happens as a function of stimulus strength. The human experiment results are intriguing, and the model seems to capture very fine details of the experiment.

Although I like the paper, I think that there are some aspects of it that deserve deep attention by the authors”.

**[C1-1a]** “First, humans are faced with 5 stimulus values, but the model does not seem to use this fact at any point (Is the distribution over stimulus values assumed to be a discrete?). To be honest, I am not able to see what the prior distribution over the biases (B) is, or how it evolves over trials (no example is provided). This is important, because inference about the mean of the distribution will strongly depend on the assumed distribution over the stimulus strengths that the participants see. In particular, how does it affect the fact that there is a maximum strength value that can be ever observed? My intuition says that the model does not take this into account, but then I would not be sure about whether the bias reverses with stimulus strength in a model where inference is based on the knowledge that there is a maximum stimulus strength. In general, the description of the model is quite confusing, mostly because of the lack of explicit equations for some basic quantities.”

**[Our reply to C1-1a]** We agree with the reviewer's comment that we should have provided a clearer description of the proposed model, BMBU. Specifically, we should have explicitly explained (i) how BMBU incorporates the use of five distinct stimuli in the experiment, (ii) how the prior distribution of the class boundary $B$ is defined and updated over trials, and (iii) how (i) and (ii) relate to the “stimulus-dependent bias reversal,” the hallmark feature of BMBU.

As pointed out by the reviewer, the above three issues stem from the unclear model description in the original manuscript. To address them, we have completely rewritten the subsection of the **Materials and methods** that describes BMBU (titled “**World-updating model**”). Specifically, regarding (i), we clearly defined the “learned” generative model and explained how it can differ from the “actual” generative model, with a rationale and references (read the first paragraph of the subsection titled **“Learned” generative model**). We have provided explicit definitions for the stimulus distribution conditioned on $B$ (Equation 3). As for the issue of (ii), we have specified how $B$ is updated over trials (Equations 13,14). Since the issue of (iii) is rooted in the issues of (i) and (ii), the above revisions addressing (i) and (ii) naturally entail and resolve the issue of (iii). Below, as our direct and integrative reply to **[C1-1a]**, we will provide a recap of how we dealt with the three issues mentioned above in the revised manuscript.

Regarding issue (i), BMBU assumes that the decision-maker “has learned” how its noisy sensory and mnemonic measurements ($m$ and $m’$), external stimuli ($S$), and true class states ($CL$) relate to one another through the latent variable $B$. Importantly, this “learned” generative model can differ from the “actual” generative process. Particularly, following a convention of Bayesian modeling work on the PDM literature (Ma, 2019), BMBU posits that $S$ is considered a random variable that is sampled from a “continuous” distribution, which is centered around $B$ with a certain width $\sigma_{S}^{2}$ in the learned generative model, due to the noises in the human brains’ sensory and working-memory systems. This is why BMBU approximates the generative process relating $S$ and $B$ with a Gaussian function ($p\left( S | B \right)=N(S;B, \sigma_{S}^{2})$); Equation 3 in the revised manuscript), despite the fact that the experimenter presented human observers with 5 discrete stimuli.

Regarding issue (ii), BMBU assumes that the decision-maker continually updates their belief about the location of $B$ (i.e., where the center of the stimulus distribution is in the size dimension) from a “prior” belief to a “posterior” belief by incorporating the information gleaned from the current PDM episode in the “likelihood” of $B$ given two pieces of evidence, $m_{t}^{'}$ and ${CL}_{t}$. Here, following the dynamic Bayesian framework, the “prior” belief in the current trial ($p(B_{t})$) is inherited from the “posterior” belief in the previous trial ($p(B_{t-1}|m_{t-1}^{'},{CL}_{t-1})$), as defined in Equations 13 and14, while the “likelihood” of $B$ in the current trial ($p(m_{t}^{'},{CL}_{t}|B_{t})$) refers to the decision-maker’s probabilistic inference about $B_{t}$ based on the information available in the current PDM episode (the noisy memory recall of the sensory measurement retrieved in working memory, $m_{t}^{'}$, and the state of the class variable ${CL}_{t}$informed by the feedback to the choice), as derived in Equations 8-12. As suggested by the reviewer, we also added a figure (S2 Fig in the revised manuscript) to visually illustrate how $B$ evolves through the PDM episodes experienced over an example sequence of trials, to help readers understand how $B$ is updated over trials.

---------------------------------------------------------------------------------------------------------------------

**S2 Fig. Example trial courses of estimated class boundary. (A)** An example trial history to show how a temporal trajectory of the class boundary inferred by BMBU. For example, at trial #1 (x-axis), a physical stimulus (symbol x) was 0, a sensory measurement (symbol o) was a positive value when the boundary belief (solid black bar; y-axis) was centered at 0. BMBU’s choice was $large$ (symbol square on the top of y-axis), and correct feedback (same square filled with green color) was provided, which indicates that the class variable at trial #1 ${CL}_{1}$ was $large$ (arrow’s direction indicates the effect of the trial class variable on the subsequent boundary-updating). BMBU updates one’s belief based on evidence from stimulus (colored symbol o) and feedback (${CL}_{1}$), available at the time of boundary-updating. To illustrate cases where the bias reversal we defined in Fig 3D in the main text happen and do not happen, same examples were intentionally used as those we used in S1 Fig where we further detailed on the model’s mechanisms. Depending on colors, sensory evidence is weak (yellow symbol o) or strong (purple symbol o), which leads to whether or not the reversal happens. Trial cases featured in a red box indicates that the “Reinforcement” principle is held (predicting subsequent choices to repeat $large$ choice) while those featured in a green box indicates that the “Reversal” happens (predicting subsequent choices to reverse the previously made $large$ choice). **(B)** Temporal trajectories of the class boundary when the same 6-trial sequence of physical stimuli in **(A)** was simulated for 100 times. This means that different $m$ and $m^{'}$ were realized.

---------------------------------------------------------------------------------------------------------------------

Regarding issue (iii), the extent to which $B$ is updated to be attracted towards the stimulus (its mnemonic measurement $m’$, to be exact)—and thus how strongly the stimulus-dependent bias reversal occurs—is affected jointly by all the parameters in BMBU ($\theta=\{\mu_{0}$ (static bias of $B$)$,\sigma_{0}$(how weak the static bias of $B$ is)$, \sigma_{m}$(sensory measurement noise)$,\sigma_{S}$(how widely stimuli are distributed—i.e., stimulus prior width), $\sigma_{m^{'}}$ (working memory noise)$,\sigma_{diffusion}$(how diffusive the working memory becomes over time)$\}$), which were set free to capture the individual differences in statistical details across their respective “learned” generative models. Nonetheless, when it comes to how (i) and (ii) relate to (iii) —the reviewer’s concern, it is important to understand how $\sigma_{S}$(how widely stimuli are distributed) affects the extent to which $B$ is updated toward the external stimulus (its mnemonic measurement $m’$, to be exact). In a nutshell, the greater $\sigma_{S}$is, the less the impact of the stimulus on $B$-updating is. This is because the likelihood function of $B$ becomes broad (less certain) as $\sigma_{S}$increases (see Equation 12), which eventually makes sensory evidence contribute less to the posterior belief of $B$. In other words, for the decision-makers with a widely distributed stimulus prior in their learned generative models, the stimulus-dependent bias reversal is more likely to be weak (as shown in Reply Fig 1B,C below).

---------------------------------------------------------------------------------------------------------------------

**Reply Fig 1. Stimulus knowledge assumed in our model (i.e. unbounded stimulus assumption) reasonably reflects actual stimuli used during the experiment, and bias reversal does not result from the unbounded stimulus assumption. (A)** Blue histogram (upper panel): A participant’s assumption about possible physical stimuli during the experiment if one had known that they had bounded ranges. For visualization, a uniform distribution $U\left( -2.5, 2.5 \right)$ is used. Dotted line (upper panel): a descriptive fit to occurrences of stimuli with a Gaussian distribution. Under this hypothetical assumption, a noise parameter for stimulus is 1.44 (denoted by $\sigma_{S, physical}$). White histogram (lower panel): Participants’ recovered stimulus noise parameters $\sigma_{S}$from BMBU model fitting. Black line (lower panel): Location of $\sigma_{S, physical}$. **(B)** Illustration of BMBU’s boundary-updating. Depending on $m’$, the “bias reversal” do or do not happen. **(C)** Depending on $\sigma_{S}$, the same $m’$ that resulted in the reversal (the case featured in purple color in **(B)**) may not cause the reversal. It should be noted that higher noise in $\sigma_{S}$ is more likely to weaken the stimulus-dependent bias reversal.

---------------------------------------------------------------------------------------------------------------------

Given the link between the stimulus prior width ($\sigma_{S}$) and the stimulus-dependent bias reversal described above, one may also wonder how closely the fitted values of $\sigma_{S}$of BMBU reflect the actual range of the stimuli used in the experiment ($\sigma_{S, physical}$). To be specific, one could ask whether participants’ stimulus prior widths were fitted as unrealistically large values, allowing for stimuli to be generated far outside the physical range of stimuli ([-2 2]). To check the correspondence between the learned and actual generative models in stimulus prior width, we approximated the value of $\sigma_{S, physical}$by determining the Gaussian function’s width that closely corresponds to the actual (uniform-like) distribution of stimuli (as depicted by the blue histograms in the top panel of Reply Fig 1A), which we found to be 1.44. As summarized by the bottom histogram in Reply Fig 1A, the fitted values of $\sigma_{S}$in most participants turned out or gather around the value of 1.44. These results ensure that the stimulus distributions in the “learned” generative models posed by BMBU reasonably well correspond to the actual stimulus distribution.

**[C1-1b]** “If there are only 5 stimulus values, why participants not just memorize them and simplify the computation of what is large and small? In particular, confidence would be very low when stimulus strength is zero, but this is not tested in the experiments, nor in the model.”

**[Our reply to C1-1b]** The reviewer proposed that human observers may rely on memorized discrete states of stimuli when making decisions instead of using inferred probabilistic beliefs about the stimulus and boundary, as suggested by BMBU. While we acknowledge that this "discrete-stimulus-memory-based" scenario is a possible alternative strategy due to its simplicity and low computational cost, we believe it is unlikely that the observers in our experiment actually adopted this strategy for several reasons. Below, we’ll describe this alternative strategy of performing binary classification that was proposed by the reviewer (, which will be referred to as the ‘discrete-memory strategy,’) and then present evidence against this strategy.

It should be noted that the discrete-memory strategy requires observers to be aware of the presence of discrete stimuli in the experiment. With noisy sensory evidence of the stimulus, the observers will categorize it into one of the discrete memory templates, such as {XS, S, M, L, XL} (in case they know there are five discrete sizes). They will then translate these discrete memories into binary decisions by mapping XS and S to 'small choice,' L and XL to 'large choice,' while making stochastic coin-flipping decisions between 'small choice' and 'large choice' for M. As a result, the decision confidence, which reflects the distance between M and a given discrete memory template, will only have three discrete values under this strategy (XS and XL for high confidence levels, S and L for intermediate confidence levels, and M for the lowest confidence level). Reply Fig 2A illustrates the discrete-memory strategy and its discrete levels of confidence accompanying decision-making.

---------------------------------------------------------------------------------------------------------------------

**Reply Fig 2. RT data favors inference scenario rather than memorization scenario. (A)** Stimulus memorization scenario. An observer does not perform perceptual inference, instead maps each of 5 stimuli into the respective stimulus values, making binary choices. In the case of the stimulus value of 0, the observer’s uncertainty is highest compared to those for the other values (see the Observer’s knowledge box). **(B)** Under memorization scenario, uncertainties are predicted around discretized clusters (multimodality). **(C)** Under inference scenario, uncertainties are predicted with smooth continuity (unimodality) **(D)** Top: Statistical test results on the unimodality of the distributions of uncertainty values predicted under each scenario (Memorization vs. Inference). Distributional line fits to bars of uncertainty values are overlaid. Vertical lines indicate each mean of the distributions for different stimulus values (dotted in black: +2/-2, solid in dark gray: +1/-1, solid in light gray: 0). Bottom: Results on the distributions of each individual’s RT data (n=30). **(E)** Two panels on the left: Accuracy sorted by sensory evidence, and further by RT (black: faster RT trials half-split, gray: slower RT). Two panels on the right: RT sorted by sensory evidence and correctness (green: correct trials, red: incorrect trials).

---------------------------------------------------------------------------------------------------------------------

It is unlikely that the observers in our task adopted this strategy for several reasons. First, as mentioned in our previous reply to **[C1-1a]**, the observers in our experiment were not informed about the number of stimuli presented during the experiment. Additionally, we conducted post-experiment interviews which confirmed that they were unaware of the fact that a discrete number of stimuli were actually used. As a result, it is unlikely that they will utilize the discrete-memory strategy as they will not arbitrarily attempt to categorize their sensory experiences into discrete memory templates without being certain of the presence of discrete stimuli. Such an effort would take away cognitive resources, making it an unlikely approach for the observers in our experiment.

Second, the discrete-memory strategy implies that the observers form discrete levels of decision confidence, but the observed distributions of response time (RT, a well-known behavioral (reciprocal) proxy of decision confidence in PDM tasks) do not appear to support that prediction. To quantitatively demonstrate this, we simulated the discrete-memory strategy for 10,000 trials by assuming that the decision-makers sort their sensory measurements ($m$) into one of the five discrete memory templates. As summarized in Reply Fig 2B, there are only three fixed levels of confidence (as depicted by the histograms) regardless of stimulus size. To be sure, these discrete distributions of confidence will be translated into smoother distributions of RTs due to motor errors (as depicted by the curves overlaid on the histograms). Thus, RT distributions are likely to exhibit a (same) discrete number of modes regardless of the stimulus size presented (the top left panel of Reply Fig 2D). However, the observed distributions of RT did not show a discrete number of modes (the bottom of Reply Fig 2D). Instead, they were best described as having a single mode, which differed in location across the stimulus conditions. These results are close to what is predicted by BMBU (the top right panel of Reply Fig 2D). Unlike the discrete-memory strategy, the decision uncertainty (reciprocal of decision confidence) takes continuous values in BMBU because it reflects the extent to which the probabilistic belief distribution of B(oundary) and that of S(timulus) are overlapped (as depicted in Reply Fig 2C). Such probability-based definition of decision confidence is known to account for the systematic relationship between decision accuracy, sensory evidence strength, and RT. Consistently, our data also exhibit such relationships, as depicted in Reply Fig 2E.

Lastly, the discrete-memory strategy does not seem to provide any principled account for the stimulus-dependent bias reversal, the target phenomenon we seek to explain. For that matter, the discrete (stable) nature of memory templates makes it hard to expect the observers to display any dynamic sequential effects (history effects) unless more assumptions are added, which would make the strategy more complex.

In sum, the observers in our experiment were unlikely to use the discrete-memory strategy for the reasons mentioned above. To be sure, the discrete-memory strategy could be effective in certain situations where the use of discrete stimuli is explicitly known. However, investigating whether such a scenario works under what conditions is certainly beyond the scope of our current work. Our focus is on determining which of the two contending scenarios of corrective-feedback learning—the value-updating and world-updating scenarios—can better explain the feedback effects observed in consecutive PDM episodes. We kindly request that the reviewer understands our decision to only share these points in this response and not include them in the revised manuscript.

**[C1-2]** “Second, I wonder how general the results are. In many PDM tasks, the boundary is very natural and intuitive: a vertical line boundary and detect whether stimulus is moving to the right or to the left respect to it. Is there any evidence that boundaries that hold some symmetries are also subject to learning?”

**[Our reply to C1-2]** We thank the reviewer for directing our attention to this issue, which is worthy of discussion as it relates to the extent of the stimulus-dependent bias reversal's generalizability.

Any given binary classification task falls somewhere in a spectrum spanning the degree to which the class boundary is arbitrary—thus more requiring learning—or explicit—thus less requiring learning. Our study and another human study cited (Hachen et al., 2021) in our paper fall on one end of the spectrum, where the class boundary is arbitrary as it is context-dependent in the sense that there is no predetermined boundary to determine whether a given stimuli is $small$ or $large$. On the other end of the spectrum is a type of binary classification task where an explicit reference stimulus is given in a separate interval for direct comparison (so-called “two-interval discrimination task”). For example, in another human study cited in our paper (Urai et al., 2017), observers judged whether the target motion stimulus was more or less coherent than the reference motion stimulus. Other studies have an implicitly defined class boundary that still seems to require some degree of learning. For instance, in an animal study cited in our paper (Lak et al., 2020), rats classified odor-mixture stimuli that vary in mixture ratio into two classes, where the class boundary is “normatively” defined as a perfectly balanced mixture stimulus (e.g., odor A 50% + odor B 50%).

As we discussed in the previously submitted version of our manuscript (specifically the second paragraph of the **Discussion** section; S7A,B Fig), we found the stimulus-dependent bias reversal in all the three studies mentioned above (Hachen et al., 2021; Lak et al., 2020; Urai et al., 2017). This suggests that the stimulus-dependent bias reversal appears generalizable to other binary classification tasks with varying degrees to which class boundary is arbitrary or explicit. However, we are cautious in making any definitive conclusions as further empirical testing is required. We discussed this matter in the revised manuscript's second paragraph of the **Discussion** section.

**[C1-3]** “I could not see any difference between Figs. 4 and 5. Are they identical?”

**[Our reply to C1-3]** We erroneously uploaded the same figure for both Figs 4 and 5. We are deeply sorry for the inconvenience this may have caused due to our oversight. We have rectified this error by uploading the appropriate figure files in the revised manuscript. Moreover, we have made further enhancements to the figures to ensure their clarity.

**[C1-4]** “Several important works on slow fluctuations and role of feedback in biases and behavior are missing and are relevant for the introduction and discussion:
Prefrontal cortex represents heuristics that shape choice bias and its integration into future behavior – ScienceDirect https://www.nature.com/articles/ncomms14823”

**[Our reply to C1-4]** We appreciate the reviewer for bringing to our attention the Moreno-Bote group’s important studies on the history effects on PDM. We recognize the significance of these studies in relation to our work. We cited and discussed them in the **Introduction** and **Discussion** sections of the revised manuscript.

Nogueira et al. (2017)’s work is relevant to our research in three ways. Firstly, it adds to previous evidence of feedback’s impacts on PDM, which we cite in the opening paragraph of the **Introduction** section, where those efforts are cited. Secondly, this study deals with multiple history factors concurrently, such as ‘stimuli,’ ‘choice,’ ‘reward,’ highlighting the importance of the multiplexed nature of the history effects in PDM. We referenced this study along with others in the third paragraph of the **Discussion** section. Lastly, we make a note of the close relationship between Nogueira et al. (2017)’s “second-order prior” with the “true state of the class in our study, which is jointly informed by ‘choice’ and ‘feedback (reward outcome).’ We mention this in the third paragraph of the **Discussion** section.

Mochol et al. (2021)’s work is also relevant to our work. They noted that there is a slow fluctuating component in choice bias in PDM and attempted to differentiate it from the bias arising on a trial-to-trial basis by taking a regression approach. We included their work in our discussion of previous efforts to establish effective methods for probing behavioral data for the multiplexed history effects in the last paragraph of the **Discussion** section.

**[C1-5]** “Given that the number of parameters is not very large compared to the number of trials, it is unlikely that adding lapses in the fitting would introduce overfitting, which means that it would be desirable to use lapses rate to better estimate biases and avoid any possible confound.”

**[Our reply to C1-5]** In our original manuscript, we made a conscious decision not to include the lapse (or guess) parameters in our PSE (our bias measure). We did this to prevent any potential overfitting problems for PDM episodes that occur infrequently, resulting in a small number of trials. However, we understand the reviewer's concern that omitting lapse parameters could cause confounding problems if episode types, such as stimulus difficulty, correlate with lapses. To address this concern, we conducted PSE estimation procedure once more, this time by incorporating the lapse and guess rates as free parameters. We found that the results did not differ between the original estimation procedure without lapse or guess rate (as shown in Reply Fig 3A identical to Fig 7A in the main text) and the modified procedure with lapse and guess rates (Reply Fig 3B). Accordingly, we revised the subsection of **Materials and methods** titled “**Data Analysis**.” In doing so, we provided further details in the PSE estimation procedure (Bootstrap-related one).

---------------------------------------------------------------------------------------------------------------------

**Reply Fig 3. Identical results of PSE estimation. (A)** The original PSE results (identical to Fig 7A in the main text). **(B)** PSE estimated when lapse and guess rates were included. They showed no statistical difference (P=0.2023 ~ 1.000, paired two-tailed *t*-tests with Bonferroni corrected).

---------------------------------------------------------------------------------------------------------------------

**[C1-6]** “I do not fully understand the role of m' and m. As m is both hidden to the experimenter and to the agent, I would recommend removing it from the model, unless I am missing something important that makes both of them necessary.”

**[Our reply to C1-6]** As pointed out by the reviewer, positing an additional noisy process ($m\to m'$) may sound redundant. However, this generative process is necessarily required for the Bayesian inference for boundary-updating in BMBU. Unlike the inference for decision-making, which relies on the sensory measurement ($m$), that for boundary-updating cannot rely on the same sensory measurement but must rely on the mnemonic measurement ($m'$) because the evidence for the stimulus is no longer available at the moment of boundary-updating, which occur at least after feedback is revealed because the boundary inference requires the combined evidence for the stimulus and the class variable. In principle, removing the process of relating $m$ to $m'$ means assuming that working memory is perfect (noise-free). However, that assumption is highly implausible because it has been well established that working memory for continuous visual features are known to be degraded very quickly (Bays et al., 2011; Wilken & Ma, 2004). In addition, as pointed by a previous BDT modeling work on PDM (Luu & Stocker, 2018), an additional (random) sampling process associated with this $m$-to-$m’$ generative process adds a dynamic quality to a consecutive pair of inferential acts (e.g., $m$ falls on the small side but can be followed by $m’$ falling on the other side), which is known to be required to capture the dynamics of inferential behavior. For the same reason, recent modeling studies built upon Bayesian Decision Theory (Lee et al., 2023; Luu & Stocker, 2018, 2021) like our study all incorporated the noisy generative process of relating $m$ to $m'$. These studies also showed that the model-fit levels of memory noise are well above zero (i.e., $\sigma_{m^{'}}^{2}\gg0$) and there are substantial degrees of individual differences accounting for individual differences, which are also observed in our study.

We regret that we did not sufficiently explain why we had to introduce the noisy mnemonic process in BMBU in the original manuscript. The rationale for this process is added to the revised manuscript, along with the references mentioned above (see the **Materials and methods’** subsections titled “**“Learned” generative model”** and **“Boundary-updating”** under **“World-updating model”**).

**[C1-7]** “The inequalities in Eq. of line 648 are very tricky, as the stimulus strengths are discrete. What happens when B=0?”

“Eq of Line 654, I guess that CL here refers only to "small". Otherwise, I do not see how it would be correct.”

“Line 657. "for simplicity" no, you define quantities in this way.”
“Eq of 661 is not identical to the previous equation in line 655?”

**[Our reply to C1-7]** In response to the reviewer's comments and questions, we have reorganized the equations related to the computation of boundary likelihood and provided more details on how they were derived. Specifically, we have formalized the boundary likelihood inference using five equations (Equations 8-12) and explained how they are related to the equations that formalize the "learned" generative model (Equations 3-6). In addition, to help readers intuit from this formalism the key behavior of BMBU (i.e., how it infers the boundary likelihood from the mnemonic evidence for the stimulus ($m'$) and the informed state of the class variable (${CL}_{t}$)), we have elaborated further on how Equation 9 is reduced to Equation 10 by providing additional equations and figure panels (Text in S1 Appendix and S1 Fig). Below, we have provided direct answers to the reviewer's questions, respectively.

Regarding the question about the equation in line 648 in our original manuscript (

“The inequalities in Eq. of line 648 are very tricky, as the stimulus strengths are discrete. What happens when B=0?”), we were a bit puzzled by what the reviewer meant by “B=0?”. If the question pertains to the discrete nature of the stimuli used in the experiment, we want to reply that the 0 value of B (B=0) is not different from any other values of B because S is assumed to be continuous (not discrete) as we clarified in **[Our reply to C1-1a]**. Alternatively, if the question refers to “B=S”, the reviewer’s confusion was probably caused by the fact that the right-hand side of the equation above did not cover the case for “B=S”. In the revised manuscript, we replaced the above equation with Equation 9, which covers the full range of the variable marginalized over (S) with two integrals. As mentioned above, we also explained in detail how Equation 9 is reduced to Equation 10 when conditioned on the informed state of the class variable (${CL}_{t}$) by providing Text in S1 Appendix (Equations S1-S6).

Regarding the question about the equation in line 654 in our original manuscript (

“Eq of Line 654, I guess that CL here refers only to "small". Otherwise, I do not see how it would be correct.”), the reviewer's observations regarding the equation are accurate. It was dependent on the condition where “$CL=small$”. To avoid any confusion, we have defined the equations separately based on the class variable's state (“$CL=small$” and “$CL=large$”) in the revised manuscript (Equations 10-12). Regarding the matter at hand, it has come to our attention that there were typos in defining the standard deviations of Gaussian distributions in lines 655, 661, and 644 of the original manuscript. In the revised manuscript, the typos have been corrected as they now appear in Equations 11 and 12.

Regarding the question about the phrase in line 657 (“Line 657. "for simplicity" no, you define quantities in this way.”), the reviewer is right. We removed the phrase in the revised manuscript (the line following Equation 11).

Regarding the question about the phrase in line 661 (“Eq of 661 is not identical to the previous equation in line 655?”), the reviewer's observation regarding the equation is accurate. As mentioned above, to avoid confusion, we have showcased the equations separately based on the class variable's state (“$CL=small$” and “$CL=large$”) in the revised manuscript (Equations 10-12).

**[M1-1]** “Minor: Line 49: "The RL algorithm". RL refers to a set of algorithms, not to a single algorithm.”

**[Our reply to M1-1]** We replaced “The reinforcement-learning (RL) algorithm” with “Reinforcement-learning (RL) algorithms.”

**[M1-2]** “Line 87. Unclear what the "stronger stimulus strength" means. Later it is used "for strong evidence", but all that seems to contradict each of the sentences. Please, clarify. Overall, that passage is not very clear, and more intuition might be needed.”

**[Our reply to M1-2]** We regret that our inconsistent use of "stimulus strength" and "strong sensory evidence" in our previous manuscript could have caused confusion. In the revised version, we have clarified the terminology and consistently used "strong sensory evidence" throughout the text. We have made significant changes to the Introduction section to help readers better understand the differences between value-updating and world-updating scenarios, particularly regarding how the strength of sensory evidence affects feedback effects. We have used more specific examples (feedback for temperature classification) to aid in comprehension.

**[M1-3]** “Line 237. Clarify that "choice" refers to the "next choices"”

“Line 238 and following one. It would help to clarify how changes of B are related to changes of PSE.”

“Line 239. It is unclear what "the size of 0 will hardly move…" means.”

**[Our reply to M1-3]** We addressed the reviewer’s points by making substantial changes to the Results subsection titled **“The Bayesian model of boundary-updating (BMBU)”** because we believe that those points stem from the limited description of why and how BMBU predicts the “stimulus-dependent bias reversal”. Consequently, we expanded this subsection from three to five paragraphs. Furthermore, since we have made significant changes to the **Materials and methods** section in our response to **[C1-1a]** (refer to **[Our reply to C1-1a]**), we needed to incorporate those modifications in the Results section as well.

**[M1-4]** “Line 629. This last sentence is repetitive with respect to the next one.”

**[Our reply to M1-4]** In response to **[C1-1a]** (refer to **[Our reply to C1-1a]**), we have made significant changes to the **Materials and methods** subsection titled “**World-updating model**.” We incorporated the reviewer’s **[M1-4]** suggestion in that revision.

**Comments from Reviewer #2:**

“In this paper the authors seek to determine whether models that cast perceptual choice updating in response to feedback as a process of learning the value associated with each alternative as opposed to learning the statistics of the environment. This is an interesting question because it touches on the fact that while animals learn perceptual decision tasks through a process of reinforcement learning, humans are trained in a very different way. Behavioural data were collected from participants performing a task in which rings had to be classed as larger or smaller than the average observed distribution. The authors hypothesise that the two different models make distinct predictions for the influence of feedback on subsequent choices. Where the value-learning models would predict that 'correct' feedback following a stimulus that is far from the currently perceived category boundary will increase the probability of selecting that alternative on subsequent trials, the world statistic model makes the opposite prediction. The authors find that a bayesian implementation of the world statistics model provides a markedly superior fit to the data compared to the value-based learning model.

I think this paper can make a potentially important contribution to the literature however I am not sufficiently expert in either reinforcement learning models or bayesian modelling to evaluate the methodology. I do wonder whether the paper would be more suited to PLOS Computational Biology given the complexity of the model comparisons.”

**[C2-1]** “My one comment is that in the introduction the authors should be more clear about what is meant by 'strong sensory evidence' - do they mean a larger ring/tree? or a ring/tree that is further from the current choice boundary irrespective of size? I believe it's the former.”

**[Our reply to C2-1]** This comment resonates with a minor comment made by Reviewer #1, specifically **[M1-2]**. In our reply to this comment (see **[Our reply to M1-2]** above), we clarified the term “strong sensory evidence” by defining it as “the extent to which a given sensory measurement of the stimulus supports one of the choices.” We included this definition in the **Introduction** section of the revised manuscript. In addition, we provided a more comprehensible example (temperature classification) to further explain how the strength of sensory evidence affects feedback effects.

**Comments from Reviewer #3:**

“In a perceptual decision-making task, Lee at al. study the behavior of human subjects and show that they use evidence to update a "world model", thereby learning some sort of underlying model of the world. They bring some evidence that human behavior doesn't conform to a more popular RL-based account, which learn values from trial-and-error.

The article is well thought out: it presents an interesting question (whether humans use the statistical structure of the world for behavior) based on previously observed counter-intuitive behavioral signatures. Lee et al. show that the "world model" alternative can explain away these signatures. A big strength of the paper is the simulation analysis which enables us to understand why assuming a statistical structure allows for a better explanation of the human behavior.”

**[C3-1]** “However, this paper suffers from a serious limitation: whereas **I am convinced** **from this work that the human behavior uses** some sort of **"world model"**, **I am not convinced that "human decision-maker do not conform to [the reward-based decision-making] assumption**". I do **not see** **why the human's internal model could not be a mixture of both.** Importantly, it does not have to be implemented in the brain as a mixture of strategies to be better explained in modelling by a mixture of strategies. I see that the authors have tried to address this concern by performing a model comparison using a "hybrid" model as a candidate model. However, I found this to be rather unconvincing for 2 reasons**: 1/ why isn't the "hybrid" model included in the simulation analysis?** Only the Value and World models are included figure 7 even though the "hybrid" model is the second-best performing model (with inconclusive evidence for the AIC). 2/ the model comparison between the "World" and "Hybrid" model is rather unclear, BIC is well-known to over-penalize models with more parameters, so it is not very surprising that "hybrid" model is going to lose in the terms of BIC (in the sense that the choice of the criteria penalizes the "hybrid" model).
Generally speaking, if you use BIC and AIC as criteria, one:
- should use the BIC if you want the model with the most parameters to win (the BIC favors the model with least parameters)
- one should use the AIC if you want the model with the least parameters to win (the AIC does not favor the model with least parameters)
Here, we are in the latter case and AIC gives inconclusive results for the comparison "world" against "hybrid" models.
When using AIC or BIC criteria, it is also important to perform some model recovery procedures to verify that the models are dissociable given the experimental task, the fitting procedure and the comparison metric. Two things: 1/ if the model recovery procedure shows that the models are dissociable with the BIC criteria, then the model selection argument based on BIC would be sound.

**And 2/ to avoid the issue of having to choose between AIC an BIC**, it would be preferable to use a cross validation procedure. Holding out some sequences, fitting on the held-in sequences and testing on the held-out. This would provide a stronger argument for the world model over the hybrid model.
Also, to make this claim (world model over mixture model), it would be important to try a simpler alternative of a mixture of both strategies: (1 - w) world + w value (instead of assuming a multiplication - page 7). Likewise, to rule out RL models, it would be important to test for RL-based alternatives that tend to work better on human behavior, such as Pearce-Hall (learning rate scaling with absolute prediction error).
**If these extra tests are not performed**, the authors **would have to tone down to the claim against the "reward-based decision-making"** assumption and make it more about the presence of "statistical structure" in the behavioral strategies (rather than the absence of RL strategies)”

**[Our reply to C3-1]** We thank the reviewer (i) for making us realize that we were not sufficiently clear about the valid claim that we can make based on the entire set of our results and the exact contribution that the goodness-of-fit results (which the reviewer’s comment was made about) make to that claim and (ii) for suggesting several ways to improve the methodological rigor when comparing and validating the models.

As for (i), it is important to distinguish between (a) the claim we are allowed to make based on the goodness-of-fit results (, which the reviewer’s comment is about) and (b) the claim we are allowed to make based on the entire set of our results as a whole, including especially those from the *ex ante* and *ex post* simulations. The core of our claim is that the value-based model in principle cannot explain the “stimulus-dependent bias reversal of feedback effects (referred to as SBRF in short here)”. This claim is supported jointly by the *ex ante* and *ex post* simulations, the former showing that the value-updating model is incapable of displaying SBRF with a reasonable range of model parameters (as summarized in Figs 4 and 5) and the latter showing that the value-updating model fit to the observed choice behavior does not reproduce the phenomenon of interest (SBRF) as in the form of the observed pattern (as summarized in Fig. 7). We stress that here the goodness-of-fit results are not directly to do with SBRF. Instead, they show that the world-updating model does a better job of “predicting” the trial-to-trial variability in choice behavior when compared to the value-updating model while taking into account model complexity. We also had to carry out the goodness-of-fit analysis to identify the best-fit parameters for these two competing models of interest, which are needed to conduct the *ex post* simulation. As well pointed out by Palminteri et al. (2017), it is important to distinguish between the type of claims that are allowed to make based on goodness-of-fit results (relative model comparison results) and those based on *ex post* simulations (reproduction tests): the former “should not be viewed as definitive evidence in favor of a given model explaining **any specific phenomenon**” whereas only the latter “decide which of two hypothetical models accounts for **the behavioral effect of interest**,” where “failing to reproduce **the effect of interest** represents the model rejection criterion that falsifies one hypothesis.” Here, “**specific phenomenon**” (i.e., “**behavioral effect of interest**” or “**effect of interest**”) corresponds to SBRF in our work. In this sense, our *ex post* simulation results allow us to make the claim that the value-based model cannot account for SBRF as it fails to reproduce SBRF both qualitatively (failure of displaying the bias reversal present in the observed feedback effects, as summarized in Fig 7B) and quantitatively (significant deviations from the observed feedback effects, as summarized in Fig. 7E). We clarified the aim of our work while avoiding any over- or mis-interpretation of the goodness-of-fit results by adding an opening paragraph to the **Results’** subsection titled **“*Ex post* simulation of the feedback effects under the two scenarios”**.

Having clarified the main claim of our work as described above, we now turn to the specific issue of comparing the world-updating model and the Hybrid model, which was raised by the reviewer. We basically concur with what the reviewer wrote about this specific issue. However, we want to clarify that we are not interested in making any claim based on the comparison between these two models in goodness-of-fit performance, which only indicate how good they are in predicting the observed trial-to-trial variability in human choice behavior, which is not the main interest of our work. Instead, our interest (the goal of our study) is on the comparison between the value-updating and world-updating models. As we wrote in the original manuscript, we introduced the Hybrid model to use its performance as a hypothetically maximal level of performance that can be achieved by the two competing models of interest (the value-updating and world-updating models). We further clarified this point in the revised manuscript by rewriting the second and third paragraphs of the **Results** subsection titled “**Evaluating the two scenarios for the goodness of fit to human decision-making data**”.

As for (ii), we think that several of the methodological suggestions made by the reviewer are valid and improve the rigor of our findings, regardless of their relevance to the issue of (i). Thus, we decided to (a) present only the AIC results by getting rid of the BIC results, (b) carry out a model recovery test, and (c) show the *ex post* simulation results for the Hybrid model in the revised manuscript. Accordingly, we have revised Fig 6 for (a); added S3 Fig, a new Materials and methods subsection titled “**Model recovery analysis**”, and a new sentence in the Results subsection titled “**Evaluating the two scenarios for the goodness of fit to human decision-making data**” for (b); S5 Fig for (c). For the Hybrid model’s *ex post* simulation (S5 Fig), we also provided statistical test results where the PSE predictions of our models of interest (value-updating, world-updating, and Hybrid) were compared to human data (the Hybrid model exhibited a few conditions of significant deviation from the data while the world-updating model exhibited none; see Table D in S1 Appendix). However, regarding the reviewer’s suggestion of considering an alternative variant of the Hybrid model, we want to clarify that the suggested weighted sum variant cannot be implemented in the common platform where both the value-updating and world-updating models can be implemented. In this platform, the world-updating and value-updating algorithms are integrated via multiplicative operations to contribute to the formation of “expected value,” which respects the mathematical definition of “expected value” (the probability that a given option is correct times the value of a given option), as exemplified in the work (Lak et al., 2020). The merit of having this common platform is that the two competing models of interest can be expressed as two reduced forms of the common model, the one lacking the choice-value-updating operation (i.e., the world-updating model) and the other lacking the world-statistics(boundary)-updating operation (i.e., the value-updating model). Thus, in this common platform, the Hybrid model is naturally defined as the one where both value-updating and world-updating operations are at work. In other words, the value-updating and world-updating models are not the kinds that can be integrated with a weighted-sum formalism in our approach. We would appreciate it if the reviewer understands why we cannot consider the suggested model variant in our work.

Lastly, having addressed the issue (i) and (ii), we still believe that we need to be careful not to overclaim or overinterpret our results. Specifically, we share with the reviewer the view that human choice behavior “in general” is likely to entail both value-updating and world-updating processes. As clarified above, what we want to claim in our work is that the value-updating process is not sufficient as a principled account of the feedback effects in human PDM. Accordingly, we searched the entire manuscript for any expressions that could possibly be construed as going beyond that claim and rectified them, including the Abstract, Results, and Discussion.

**[C3-2]** “Figure 4 and 5 are identical (or at least I think) - I spent 30min trying to find differences. Am I missing something?”

**[Our reply to C3-2]** As we wrote in our reply to a previous comment from Reviewer #1 (refer to **[Our reply to C1-3]**), we erroneously uploaded the same figure for both Figs. 4 and 5. We are deeply sorry for the inconvenience. We have rectified this error by uploading the appropriate figure files in the revised manuscript. Moreover, we have made further enhancements to the figures to ensure their clarity.

**[M3-1]** “Minor: Equations in the methods are confusing. For instance, the integrals presented in the methods are integrals over probability laws (so we have an integral over a Gaussian law), I guess that it means that I should consider the probability density function and not the law but when I did that, I still didn't fully understand all the equations (and it requires efforts on the reader's side). I would recommend that the authors re-write the methods as formally as possible.”

**[Our reply to M3-1]** As we wrote in our reply to a previous comment from Reviewer #1 (**[Our reply to C1-1a]**), we have completely rewritten the subsection of the **Materials and methods** that describes BMBU (titled “**World-updating model**”). In the revised manuscript, BMBU has been described by three subsections. In the first section (“**“Learned” generative model**”), we specified the Bayesian decision-maker’s internal model for the binary classification task using a set of equations (Equations 3-6). In the following section (“**Decision-making**”), we specified how the Bayesian decision-maker infers the state of the class variable by propagating the sensory measurement over the generative model defined in the previous section (Equation 7). In the last section (“**Boundary-updating**”), we specified how the Bayesian decision-maker updates its belief about the class boundary by propagating the noisy mnemonic measurement and the informed state of the class variable over the generative model using a set of equations (Equations 8-14). In addition, we also (i) created a new figure (S2 Fig in the revised manuscript) to visually illustrate howthe class boundary is updated depending on PDM episodes experienced over an example sequence of trials; (ii) elaborated further on the derivation of Equations 9 and 10 by adding supplementary materials (refer to Text in SI Appendix and S1 Fig).

**[M3-2]** “The paragraph that introduces the "stimulus-dependent bias reversal" (last paragraph of page 9) is really hard to understand and seems to be an important paragraph. I had to look into the equations in the methods to grasp the concept.”

**[Our reply to M3-2]** We thank the reviewer for making us realize that readers may have trouble comprehending the key prediction of BMBU, “the stimulus-dependent bias reversal of feedback effects”. In response to this concern, we have significantly modified the **Results** subsection titled “**The Bayesian model of boundary-updating (BMBU)**” by expanding it from three to five paragraphs. Additionally, we have reworked the **Introduction** section to provide an intuitive description of the world-updating scenario based on a concrete example (temperature classification).

**[M3-3]** “Quality of figures were bad so there are some things that were hard to decipher (e.g. figure 4F)”

**[Our reply to M3-3]** We experienced technical difficulties converting the original figure files into TIFF formats, resulting in low-resolution images. We apologize for any inconvenience this has caused. In the updated manuscript, we have ensured that all figures are presented in their highest quality. We have also made improvements to Figs 4 and 5 to enhance their illustrative clarity.

**[M3-4]** “Are \sigma_m and \sigma_m' identifiable? I can understand that they have different "meanings" in the model but in the fitting procedure it seems like we are adding an unnecessary parameters and the only thing that we can fit is (\sigma_m)^2 + (\sigma_m')^2.”

**[Our reply to M3-4]** The reviewer’s comment resonates with a previous comment by Reviewer #1 (**[C1-6]**). Please refer to our reply to that comment (**[Our reply to C1-6]**) to see how we have addressed this issue.

**[M3-5]** “Why is the \sigma_diffusion parameters necessary? It makes the inference over B sub-optimal, what happens if you don't assume it and you assume in the exact inference for B?”

**[Our reply to M3-5]** In BMBU, the diffusion parameter was required when (imperfectly) inheriting the posterior belief about the class boundary from the previous trial to form the prior belief in the current trial. Without this parameter (when the \sigma_diffusion is set to 0, in other words), the uncertainty (width) of the belief about the class boundary would become increasingly low (narrow), being quickly stabilized into a steady point (as shown below in Reply Figure 4). This property is shared all across any dynamic Bayesian models, including Kalman filter models. For example, Daw et al. (2006)’s Kalman filter model introduced the diffusion parameter when inheriting the posterior belief from the previous trial to the current trial, similar to what was done in BMBU.

---------------------------------------------------------------------------------------------------------------------

**Reply Fig 4. Trial trajectories of BMBU’s estimates of class boundary. (A)** 5 example simulation runs are shown. **(B)** Example trajectories are shown when simulations were run with diffusion noise parameter set at 0.

---------------------------------------------------------------------------------------------------------------------

**References (for the Authors’ Responses to Reviewers)**

Bays, P. M., Gorgoraptis, N., Wee, N., Marshall, L., & Husain, M. (2011). Temporal dynamics of encoding, storage, and reallocation of visual working memory. *Journal of Vision*, *11*(10), 6–6. <https://doi.org/10.1167/11.10.6>

Daw, N. D., O’Doherty, J. P., Dayan, P., Seymour, B., & Dolan, R. J. (2006). Cortical substrates for exploratory decisions in humans. *Nature*, *441*(7095), 876–879. <https://doi.org/10.1038/nature04766>

Hachen, I., Reinartz, S., Brasselet, R., Stroligo, A., & Diamond, M. E. (2021). Dynamics of history-dependent perceptual judgment. *Nature Communications*, *12*(1), 6036. <https://doi.org/10.1038/s41467-021-26104-2>

Lak, A., Hueske, E., Hirokawa, J., Masset, P., Ott, T., Urai, A. E., Donner, T. H., Carandini, M., Tonegawa, S., Uchida, N., & Kepecs, A. (2020). Reinforcement biases subsequent perceptual decisions when confidence is low, a widespread behavioral phenomenon. *eLife*, *9*, e49834. <https://doi.org/10.7554/elife.49834>

Lee, H., Lee, H.-J., Choe, K. W., & Lee, S.-H. (2023). Neural Evidence for Boundary Updating as the Source of the Repulsive Bias in Classification. *The Journal of Neuroscience*, *43*(25), 4664–4683. <https://doi.org/10.1523/jneurosci.0166-23.2023>

Luu, L., & Stocker, A. A. (2018). Post-decision biases reveal a self-consistency principle in perceptual inference. *eLife*, *7*, e33334. <https://doi.org/10.7554/elife.33334>

Luu, L., & Stocker, A. A. (2021). Categorical judgments do not modify sensory representations in working memory. *PLoS Computational Biology*, *17*(6), e1008968. <https://doi.org/10.1371/journal.pcbi.1008968>

Mochol, G., Kiani, R., & Moreno-Bote, R. (2021). Prefrontal cortex represents heuristics that shape choice bias and its integration into future behavior. *Current Biology*, *31*(6), 1234-1244.e6. <https://doi.org/10.1016/j.cub.2021.01.068>

Ma, W. J. (2019). Bayesian Decision Models: A Primer. *Neuron*, *104*(1), 164–175. <https://doi.org/10.1016/j.neuron.2019.09.037>

Nogueira, R., Abolafia, J. M., Drugowitsch, J., Balaguer-Ballester, E., Sanchez-Vives, M. V., & Moreno-Bote, R. (2017). Lateral orbitofrontal cortex anticipates choices and integrates prior with current information. *Nature Communications*, *8*(1), 14823. <https://doi.org/10.1038/ncomms14823>

Palminteri, S., Wyart, V., & Koechlin, E. (2017). The Importance of Falsification in Computational Cognitive Modeling. *Trends in Cognitive Sciences*, *21*(6), 425–433. <https://doi.org/10.1016/j.tics.2017.03.011>

Urai, A. E., Braun, A., & Donner, T. H. (2017). Pupil-linked arousal is driven by decision uncertainty and alters serial choice bias. *Nature Communications*, *8*(1), 14637. <https://doi.org/10.1038/ncomms14637>

Wilken, P., & Ma, W. J. (2004). A detection theory account of change detection. *Journal of Vision*, *4*(12), 11. <https://doi.org/10.1167/4.12.11>

**Below, we have copied and pasted the entire review in its original form as we received it.**

________reviews start_________________________________________________________________

REVIEWS:

**Reviewer #1:**

The authors investigate the origins of correcting feedback in perceptual decision making by using computational modelling and human experiments. When comparing the predictions of a value-based model vs a world's inference model, they find that the second type explains the data much better. Most importantly, the two models different by the type of qualitative predictions that they make: the world's inference model is the only one that can explain a bias reversal that happens as a function of stimulus strength. The human experiment results are intriguing, and the model seems to capture very fine details of the experiment.

Although I like the paper, I think that there are some aspects of it that deserve deep attention by the authors.
First, humans are faced with 5 stimulus values, but the model does not seem to use this fact at any point (Is the distribution over stimulus values assumed to be a discrete?). To be honest, I am not able to see what the prior distribution over the biases (B) is, or how it evolves over trials (no example is provided). This is important, because inference about the mean of the distribution will strongly depend on the assumed distribution over the stimulus strengths that the participants see. In particular, how does it affect the fact that there is a maximum strength value that can be ever observed? My intuition says that the model does not take this into account, but then I would not be sure about whether the bias reverses with stimulus strength in a model where inference is based on the knowledge that there is a maximum stimulus strength. In general, the description of the model is quite confusing, mostly because of the lack of explicit equations for some basic quantities.
If there are only 5 stimulus values, why participants not just memorize them and simplify the computation of what is large and small? In particular, confidence would be very low when stimulus strength is zero, but this is not tested in the experiments, nor in the model.

Second, I wonder how general the results are. In many PDM tasks, the boundary is very natural and intuitive: a vertical line boundary and detect whether stimulus is moving to the right or to the left respect to it. Is there any evidence that boundaries that hold some symmetries are also subject to learning?

I could not see any difference between Figs. 4 and 5. Are they identical?

Several important works on slow fluctuations and role of feedback in biases and behavior are missing and are relevant for the introduction and discussion:
Prefrontal cortex represents heuristics that shape choice bias and its integration into future behavior - ScienceDirect
https://www.nature.com/articles/ncomms14823

Given that the number of parameters is not very large compared to the number of trials, it is unlikely that adding lapses in the fitting would introduce overfitting, which means that it would be desirable to use lapses rate to better estimate biases and avoid any possible confound.

I do not fully understand the role of m' and m. As m is both hidden to the experimenter and to the agent, I would recommend removing it from the model, unless I am missing something important that makes both of them necessary.

The inequalities in Eq. of line 648 are very tricky, as the stimulus strengths are discrete. What happens when B=0?

Eq of Line 654, I guess that CL here refers only to "small". Otherwise, I do not see how it would be correct.

Line 657. "for simplicity" no, you define quantities in this way.

Eq of 661 is not identical to the previous equation in line 655?

Minor:

Line 49: "The RL algorithm". RL refers to a set of algorithms, not to a single algorithm.

Line 87. Unclear what the "stronger stimulus strength" means. Later it is used "for strong evidence", but all that seems to contradict each of the sentences. Please, clarify. Overall, that passage is not
very clear, and more intuition might be needed.

Line 237. Clarify that "choice" refers to the "next choices"

Line 238 and following one. It would help to clarify how changes of B are related to changes of PSE.

Line 239. It is unclear what "the size of 0 will hardly move…" means.

Line 629. This last sentence is repetitive with respect to the next one.


**Reviewer #2:**

In this paper the authors seek to determine whether models that cast perceptual choice updating in response to feedback as a process of learning the value associated with each alternative as opposed to learning the statistics of the environment. This is an interesting question because it touches on the fact that while animals learn perceptual decision tasks through a process of reinforcement learning, humans are trained in a very different way. Behavioural data were collected from participants performing a task in which rings had to be classed as larger or smaller than the average observed distribution. The authors hypothesise that the two different models make distinct predictions for the influence of feedback on subsequent choices. Where the value-learning models would predict that 'correct' feedback following a stimulus that is far from the currently perceived category boundary will increase the probability of selecting that alternative on subsequent trials, the world statistic model makes the opposite prediction. The authors find that a bayesian implementation of the world statistics model provides a markedly superior fit to the data compared to the value-based learning model.

I think this paper can make a potentially important contribution to the literature however I am not sufficiently expert in either reinforcement learning models or bayesian modelling to evaluate the methodology. I do wonder whether the paper would be more suited to PLOS Computational Biology given the complexity of the model comparisons.

My one comment is that in the introduction the authors should be more clear about what is meant by 'strong sensory evidence' - do they mean a larger ring/tree? or a ring/tree that is further from the current choice boundary irrespective of size? I believe it's the former.


**Reviewer #3:**

In a perceptual decision-making task, Lee at al. study the behavior of human subjects and show that they use evidence to update a "world model", thereby learning some sort of underlying model of the world. They bring some evidence that human behavior doesn't conform to a more popular RL-based account, which learn values from trial-and-error.

The article is well thought out: it presents an interesting question (whether humans use the statistical structure of the world for behavior) based on previously observed counter-intuitive behavioral signatures. Lee et al. show that the "world model" alternative can explain away these signatures. A big strength of the paper is the simulation analysis which enables us to understand why assuming a statistical structure allows for a better explanation of the human behavior.

However, this paper suffers from a serious limitation: whereas I am convinced from this work that the human behavior uses some sort of "world model", I am not convinced that "human decision-maker do not conform to [the reward-based decision-making] assumption". I do not see why the human's internal model could not be a mixture of both. Importantly, it does not have to be implemented in the brain as a mixture of strategies to be better explained in modelling by a mixture of strategies. I see that the authors have tried to address this concern by performing a model comparison using a "hybrid" model as a candidate model. However, I found this to be rather unconvincing for 2 reasons: 1/ why isn't the "hybrid" model included in the simulation analysis? Only the Value and World models are included figure 7 even though the "hybrid" model is the second-best performing model (with inconclusive evidence for the AIC). 2/ the model comparison between the "World" and "Hybrid" model is rather unclear, BIC is well-known to over-penalize models with more parameters, so it is not very surprising that "hybrid" model is going to lose in the terms of BIC (in the sense that the choice of the criteria penalizes the "hybrid" model).
Generally speaking, if you use BIC and AIC as criteria, one:
- should use the BIC if you want the model with the most parameters to win (the BIC favors the model with least parameters)
- one should use the AIC if you want the model with the least parameters to win (the AIC does not favor the model with least parameters)

Here, we are in the latter case and AIC gives inconclusive results for the comparison "world" against "hybrid" models.

When using AIC or BIC criteria, it is also important to perform some model recovery procedures to verify that the models are dissociable given the experimental task, the fitting procedure and the comparison metric. Two things: 1/ if the model recovery procedure shows that the models are dissociable with the BIC criteria, then the model selection argument based on BIC would be sound. And 2/ to avoid the issue of having to choose between AIC an BIC, it would be preferable to use a cross validation procedure. Holding out some sequences, fitting on the held-in sequences and testing on the held-out. This would provide a stronger argument for the world model over the hybrid model.

Also, to make this claim (world model over mixture model), it would be important to try a simpler alternative of a mixture of both strategies: (1 - w) world + w value (instead of assuming a multiplication - page 7). Likewise, to rule out RL models, it would be important to test for RL-based alternatives that tend to work better on human behavior, such as Pearce-Hall (learning rate scaling with absolute prediction error).

If these extra tests are not performed, the authors would have to tone down the claim against the "reward-based decision-making" assumption and make it more about the presence of "statistical structure" in the behavioral strategies (rather than the absence of RL strategies)

Figure 4 and 5 are identical (or at least I think) - I spent 30min trying to find differences. Am I missing something?

Minor:

Equations in the methods are confusing. For instance, the integrals presented in the methods are integrals over probability laws (so we have an integral over a Gaussian law), I guess that it means that I should consider the probability density function and not the law but when I did that, I still didn't fully understand all the equations (and it requires efforts on the reader's side). I would recommend that the authors re-write the methods as formally as possible.

The paragraph that introduces the "stimulus-dependent bias reversal" (last paragraph of page 9) is really hard to understand and seems to be an important paragraph. I had to look into the equations in the methods to grasp the concept.

Quality of figures were bad so there are some things that were hard to decipher (e.g. figure 4F)

Are \sigma_m and \sigma_m' identifiable? I can understand that they have different "meanings" in the model but in the fitting procedure it seems like we are adding an unnecessary parameters and the only thing that we can fit is (\sigma_m)^2 + (\sigma_m')^2.

Why is the \sigma_diffusion parameters necessary? It makes the inference over B sub-optimal, what happens if you don't assume it and you assume in the exact inference for B?

________reviews end_________________________________________________________________
