# Supplementary material for: Corrective feedback guides human perceptual decision-making by informing about the world state rather than rewarding its choice
Source: PLoS Biol. 2023 Nov 8;21(11):e3002373. doi: 10.1371/journal.pbio.3002373 (PMC10659185; doi:10.1371/journal.pbio.3002373)
Supplement: Attachment — Submitted filename: response-to-reviewers_lee.docx [file pbio.3002373.s012.docx]

We appreciate the valuable feedback and constructive suggestions provided by the editors and reviewers on our revised manuscript. We are particularly grateful for the input on the Title, Abstract, and statistical reports. We carefully considered each suggestion and made necessary revisions to our previously submitted manuscript. We have provided a summary of our responses to the reviewers’ comments and suggestions, and we have outlined our replies to the editors' comments in our cover letter.

**Reviewer 1’s comments:** “The authors have fully addressed all my comments.”

**Reviewer 3’s comments:** “Thank you for addressing my concerns and providing a detailed response to my comments. I appreciate your clarification regarding the main claim of your work and how it relates to the goodness-of-fit results and the simulation analyses.

I also appreciate your efforts to improve the methodological rigor of your study, including using only the AIC criteria, performing a model recovery analysis, and presenting the ex post simulation results for the Hybrid model. These changes strengthen your paper and the robustness of your findings.

Regarding the suggestion for an alternative variant of the Hybrid model, I understand your explanation for why it cannot be implemented in your common platform. Given this limitation, it's reasonable to focus on the Hybrid model as defined in your approach.

Overall, your response has addressed my concerns effectively, and the revisions you've made to the manuscript enhanced the quality and clarity of your work.

One overall remark: every time a t-test is performed, the tvalue and number of degrees of freedom should be systematically reported (which is not the case here, e.g., see the "Evaluating the two scenarios for the goodness of fit to human decision-making data")”

**[Our reply to Reviewer 3’s specific comment]** As recommended by the reviewer, we have included *t*-values and degrees of freedom alongside *P*-values in all relevant sections throughout the revised manuscript.
